# The Influence of Noise Exposure on Cognitive Function in Children and Adolescents: A Meta-Analysis

**DOI:** 10.3390/neurosci6010022

**Published:** 2025-03-04

**Authors:** David Fernández-Quezada, Diana Emilia Martínez-Fernández, Ileana Fuentes, Joaquín García-Estrada, Sonia Luquin

**Affiliations:** 1 Departamento de Neurociencias, Centro Universitario de Ciencias de la Salud (CUCS), Universidad de Guadalajara (UdeG), Sierra Mojada 950, Guadalajara 44340, Mexico; 2 Instituto de Neurociencias Traslacionales, Universidad de Guadalajara (UdeG), Sierra Mojada 950, Guadalajara 44340, Mexico; 3 Departamento de Farmacobiología, Centro Universitario de Ciencias Exactas e Ingenierías (CUCEI), Universidad de Guadalajara (UdeG), Blvd. Marcelino García Barragán 1421, Guadalajara 44430, Mexico

**Keywords:** noise, cognition, cognitive function, children, adolescents, meta-analysis

## Abstract

Environmental noise has been repeatedly linked to negative effects on cognitive functioning among children and adolescents. This research sought to systematically assess studies investigating the relationship between noise exposure and cognitive outcomes in young individuals. Through a meta-analysis of eight primary studies published between 2001 and 2023, this study examined the effects of various noise types on cognitive performance across multiple domains in young populations. The findings reveal that noise exposure significantly impairs cognitive performance in children and adolescents, with a standardized mean difference (SMD) of –0.544 (95% CI: [−0.616, −0.472]), z = −14.85, *p* < 0.0001. These results underscore the profound impact of environmental noise on cognitive functioning in younger populations.

## 1. Introduction

Environmental conditions during the early stages of life are critical for the development of cognitive abilities required for future challenges in life. The environment plays a pivotal role in shaping the development of academic skills in children [1], including the development of reading skills, abstract thinking, and overall executive functions.

Environmental noise exposure is known to have a negative impact on global human health and disrupt the development of cognitive domains in children and adolescents [1]. A significant amount of work has identified that children and adolescents exposed to noise from traffic [2], aircraft [3], in home [4], or in class [5] perform worse academically and show low scores in cognitive tests, highlighting the importance of studying the impact of noise on young populations.

Both acute and chronic types of exposure to noise have an impact on cognitive performance in children and adolescents [6]. Acute classroom noise may compromise complex listening tasks: children in simulated classrooms with noise have low scores in comprehension learning tasks [7], suggesting that noise has a negative impact on the ability to process complex linguistic tasks. In highly populated cities, acute exposure to road traffic noise is often a public health concern and can negatively affect attention and overall IQ scores in elementary school children [8]. Cross-national studies can help to understand the effect of chronic environmental stressors, such as aircraft noise, on children. Research shows that areas around large airports have detrimental effects on the reading skills of elementary school students [9].

Because the frontal lobes are not fully developed until late adolescence [10], executive functions are vulnerable to noise; therefore, particular attention should be directed toward the role of noise in adolescent age. Research shows that older adolescents exposed to higher levels of in-home noise performed better than their younger peers in their ability to inhibit responses in a particular context [4], suggesting that more developed executive functions may compensate for environmentally disruptive conditions. Other cognitive skills, such as reading comprehension and vocabulary-learning tasks, can be sensitive to noise; when adolescents are exposed to higher levels of classroom noise, they show significantly lower reading comprehension skills than their peers exposed to a better acoustic environment [5]. Because the acquisition of several academic skills happens in the early stages of life, noise can have long-term effects on an individual’s daily life and negatively impact executive functions in adulthood.

Understanding the effects that noise can have on the cognitive development of the young population can help us to generate better environments for their overall health. This review aimed to systematically evaluate research examining the impact of noise exposure on young individuals on cognitive function.

## 2. Materials and Methods

This meta-analysis was officially registered with the Prospective Register of Systematic Reviews (PROSPERO) on 7 November 2024 under the identifier CRD42024606851. The search strategy for each database was designed based on the research question, following the guidelines outlined in the Preferred Reporting Items for Systematic Reviews and Meta-Analyses (PRISMA) [11].

### 2.1. Search Strategy

The search was performed across PubMed and Web of Science databases, targeting relevant research articles published from inception through 4 December 2024. Table 1 presents a summary of the keywords utilized in the search strategy, with a detailed account of the search methodology provided in Search S1.

### 2.2. Inclusion and Exclusion Criteria

We included all studies that reported the effects of noise exposure on cognitive function in children and adolescents. Only studies that employed quantitative measures of memory, learning, executive function, attention, or intelligence quotient (IQ) were selected. In cases where the abstract did not provide sufficient information for inclusion, the full text was reviewed. The analysis was limited to studies published in English. The experimental group consisted of individuals exposed to various types of noise, with the focus restricted to minors under 18 years of age.

Exclusions encompassed letters to the editor, clinical case reports, commentaries, systematic reviews, qualitative studies, short communications, and meta-analyses. Additionally, any full-text publications that were inaccessible were excluded; File S2.

### 2.3. Data Extraction

**Figure 1:**
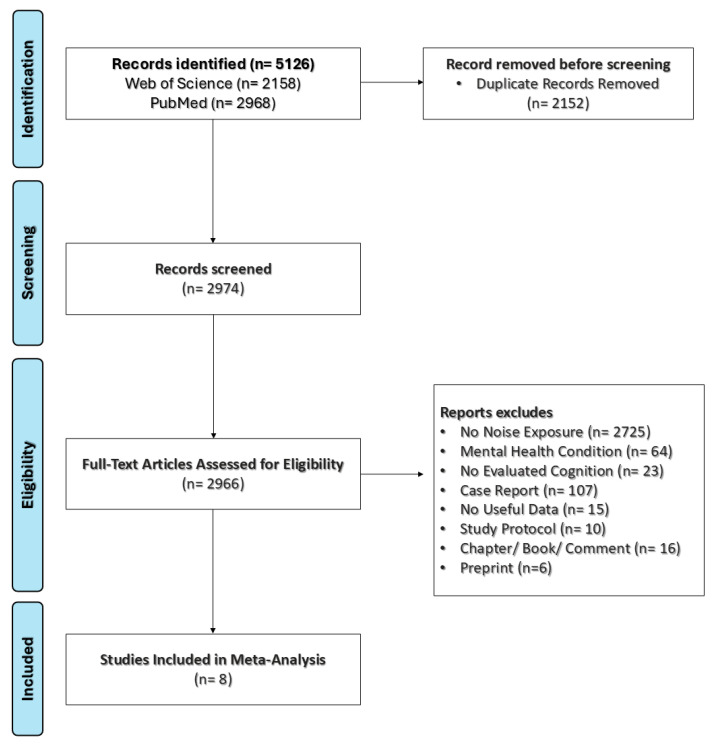
Flow diagram outlining the study selection process according to the defined inclusion criteria.

The eligible studies were screened independently and identified by the reviewers (D.F.-Q. and D.E.M.-F.) based on their titles, abstracts, and full texts, following the established inclusion criteria. After completing the selection process, relevant data from the included studies were systematically extracted and organized (Figure 1). The studies were independently coded by the researchers for key variables, including the first author’s name, publication year, journal, study design, total sample size, group-specific sample sizes, participant age, primary outcome measures, country of study, participant sex, noise source and level, methods of exposure assessment, exposure setting, cognitive outcome measures, and the primary test used to assess cognitive function.

### 2.4. Quality Evaluation

The quality evaluation of the included studies was performed using the Risk of Bias 2 (RoB 2) tool [12]. This assessment was carried out independently by two researchers, D.F.-Q. and J.G.-E., with S.L. available as an arbitrator in the event of discrepancies, though such intervention was not required due to unanimous agreement. The RoB 2 tool examines potential bias across five core domains: (1) the process of randomization, (2) deviations from intended interventions, (3) incompleteness of outcome data, (4) accuracy of outcome measurement, and (5) the selection of reported results. Each domain is evaluated based on specific signaling questions and classified into one of three categories: low risk, some concerns, or high risk. The overall risk of bias is then determined by aggregating the ratings across all domains. Studies are classified as high risk if any domain is deemed high risk or if multiple domains raise concerns. Conversely, a study is categorized as low risk when all domains are rated low risk, whereas studies with limited domains of concern fall into the intermediate category of some concerns.

### 2.5. Statistical Methods

Meta-analysis was carried out by R Software (version 4.4.2, https://www.r-project.org/ (accessed on 4 December 2024)), rmeta: Meta-Analysis (version 3.0, rmeta_3.0.tar.gz https://cran.r-project.org/web/packages/rmeta/index.html (accessed on 4 December 2024)) was employed (R code S3). We analyzed the data using random and fixed-effects models to estimate the impact of noise exposure on cognitive function between studies. The mean difference (MD) was calculated, followed by the pooled standard deviation (SD), which accounts for the shared variability between groups. Using the pooled SD, the standard error (SE) was derived by multiplying it by the square root of the sum of the reciprocals of the sample sizes from each group. To obtain the standardized mean difference (SMD), the MD was divided by the pooled SD, providing a measure equivalent to Cohen’s d, as described by Shim and Kim [13]; the extracted data are presented in Table 2. Additionally, when the mean and standard deviation were not directly available from a graph or table, these values were estimated using the methods provided by the Meta-Analysis Accelerator tool [14,15].

The I^2^ statistic was utilized to quantify the extent of heterogeneity attributable to variations in the true effect size. Effect sizes were categorized as follows: 20% indicating a small effect, 50% representing a moderate effect, and 80% reflecting a high degree of heterogeneity [20]. Forest and funnel plots were generated using the functions from the meta package.

## 3. Results

In this meta-analysis, we evaluated the impact of noise exposure on cognitive function among children and adolescents. A comprehensive database search initially yielded 5126 articles, of which eight studies from Korea, Switzerland, the United Kingdom, South Africa, and Sweden were included in the final analysis. The total sample was evenly divided between the experimental group (n = 1687) and the control group (n = 1698). The PRISMA flow diagram, presented in Figure 1, illustrates the study selection process.

### 3.1. Study Characteristics

The studies, published between 2001 and 2023, involved sample sizes ranging from 20 to 509 participants, with ages spanning 8 to 16 years. Each study included a control group that was not exposed to noise or was unrelated to noisy environments. The primary source of noise was aircraft noise, while other sources included road traffic noise, environmental noise, and white noise. Noise exposure levels ranged from 55 to 80 dB, as determined by the exposure assessments. The primary locations of exposure were schools, followed by homes.

The cognitive function in the selected studies was assessed using a variety of tests, scales, and tasks designed to evaluate distinct domains of cognition. For instance, the KIT-P assessed multiple areas of intelligence, such as verbal and non-verbal reasoning, problem-solving, memory, and processing speed, offering insights into children’s intellectual development. The IST focused on verbal, numerical, and figural reasoning, alongside memory capacity. To evaluate attentional control and executive function, the Flanker Task was utilized, specifically targeting the ability to inhibit distractions and concentrate on relevant stimuli. The Reading Task measured latency in learning, recognizing, or recalling new words during reading activities, while the KEDI-WISC assessed verbal comprehension, perceptual reasoning, processing speed, and working memory, taking into account the cultural and educational context of South Korea. Reading fluency, comprehension, and accuracy were evaluated using the SRS2, while memory function was assessed with the Verbal Episodic Recall Test. Additionally, the Suffolk Reading Scale was applied to gauge word recognition, reading fluency, and comprehension, providing a comprehensive evaluation of reading skills in children and adolescents. A summary of the characteristics of the included studies, including their cognitive assessment tools, is presented in Table 3.

### 3.2. Impact of Noise Exposure on Cognition Function in Children and Adolescents

The results revealed a significant reduction in cognition function in the youth when they were exposed to noise. Using the common-effect model, the analysis demonstrated a standardized mean difference (SMD) of −0.544, with a 95% CI [ −0.616 to −0.472], indicating a statistically significant effect (z = −14.85, *p* < 0.0001). Meanwhile, the random-effects model, which accounts for between-study variability, reported a greater SMD of −1.432, with a 95% CI [ −2.672 to −0.192] (z = −2.26, *p* = 0.0236).

**Figure 2:**
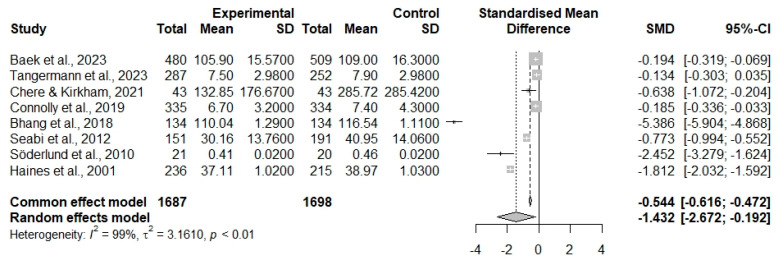
Forest plot. Negative values represent a reduction in cognitive function in the standardized mean difference.

The assessment of heterogeneity revealed high variability between the studies included in this meta-analysis, with an I^2^ = 98.3% (Q= 562.66, *df* = 7, *p* < 0.0001), Figure 2.

### 3.3. Sensitivity Analysis of Study Influence on Effect Size

To assess the robustness of the meta-analytic findings, we conducted a sensitivity analysis by iteratively excluding one study at a time and recalculating the overall standard mean difference (SMD). The range in SMDs observed during this analysis was −0.3924 to −0.7167, indicating that the overall effect size remained negative across all scenarios.

### 3.4. Quality Analysis of Research Reports

**Figure 3:**
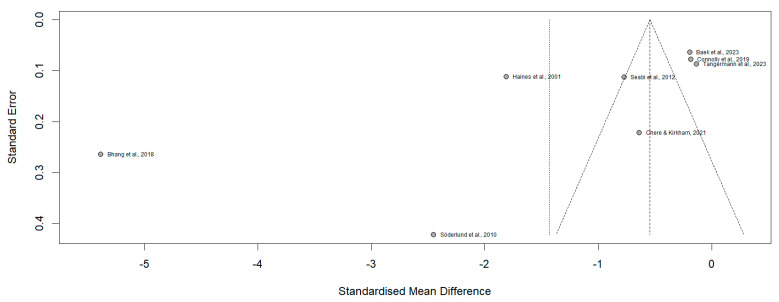
Funnel plot of the evaluated articles.

**Figure 4:**
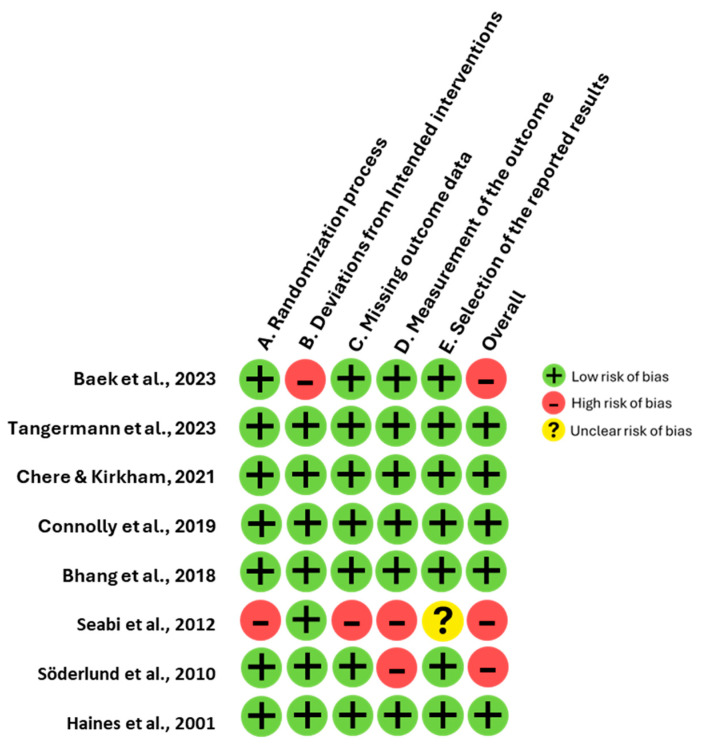
Risk of bias assessment for the eight included studies.

The funnel plot (Figure 3) demonstrates an asymmetrical distribution of studies included in the meta-analysis. Specifically, studies such as [3,8] located on the lower left quadrant, display disproportionately large effect sizes coupled with higher standard errors, suggesting potential small-study effects. Conversely, studies such as [5,16] cluster near the mean effect size with lower standard errors. Whereas [19] deviates notably towards positive values. This asymmetry indicates systematic differences in the methodologies used to measure cognitive function or in the population characteristics across the included studies. The Risk of Bias (RoB 2) assessment revealed that almost all studies demonstrated a low risk of bias across cognitive domains (Figure 4), ensuring robust methodological quality. However, refs. [16,17] showed a high risk in the randomization process and selective reporting of results, while [3] had an unclear risk due to insufficient information on missing outcome data.

## 4. Discussion

A total of 3385 children and adolescents were included in this study to assess the influence of noise exposure on cognitive function. The findings reveal that various sources of noise exposure can significantly impair cognitive abilities, including learning, memory, executive function, IQ, and other related domains. However, the analysis also identified high heterogeneity in the included studies. This suggests that the effects of noise exposure on cognitive function in youth may vary depending on study-specific factors, such as the type of noise source, population characteristics, and methodological approaches.

The high heterogeneity observed (98.3%) underscores the significant variability among the included studies. This variability could arise from several sources, including differences in the types of noise exposure (e.g., traffic noise, industrial noise, or classroom noise), variations in the measurement of cognitive function, and discrepancies in population demographics, such as age, socioeconomic status, and baseline cognitive abilities. Additionally, methodological inconsistencies, such as variations in study design (e.g., cross-sectional versus longitudinal studies) and differences in noise exposure assessment methods, may have contributed to this heterogeneity.

Moreover, the funnel plot analysis revealed an asymmetrical distribution of studies, suggesting potential small-study effects and systematic differences among the included studies. For instance, studies such as Bhang et al. [8] and Söderlund et al. [11] exhibited disproportionately large effect sizes with higher standard errors, indicating possible publication bias or methodological variability. In contrast, studies like Baek et al. [12] and Connolly et al. [5] clustered around the mean effect size with lower standard errors, reflecting greater consistency and methodological rigor. Additionally, Haines et al. [17] deviated significantly towards positive effect values, further underscoring differences in population characteristics or study design. The RoB 2 assessment confirmed overall strong methodological quality across most studies, with the majority classified as low bias. However, specific exceptions were noted. These observations emphasize the need for greater methodological transparency and standardization in future research to enhance the reliability and comparability of findings.

These findings are consistent with prior research indicating that chronic noise exposure adversely affects attention and memory processes [11,21,22]. These effects are likely mediated through stress-related mechanisms and physiological responses triggered by prolonged noise exposure [23,24,25,26]. Moreover, a large body of research in humans consistently reports that noise has detrimental impacts on cognitive performance across various domains [27].

Noise exposure impacts human physiology through direct and indirect mechanisms, both of which contribute to cognitive impairment. The direct pathway primarily involves damage to auditory hair cells caused by prolonged exposure to high sound pressure levels. This damage disrupts auditory signal processing and can indirectly affect cognitive function through impaired sensory input [28]. Additionally, noise-induced sleep disruption, a recognized cardiovascular risk factor, further exacerbates cognitive deficits by impairing neural restoration processes during sleep [2]. The indirect pathway involves emotional and metabolic responses triggered by noise stimuli. Activation of the limbic system in response to noise elicits neuroendocrine arousal, leading to alterations in glucose metabolism, lipid dysregulation, and hemodynamic changes [29,30,31,32,33,34]. These disruptions are associated with cognitive decline and a higher likelihood of developing neurodegenerative diseases. Moreover, noise levels exceeding 65 dB are known to induce oxidative damage through the production of reactive oxygen species (ROS), which significantly contribute to neuronal damage and cognitive impairment [35,36,37].

Both pathways converge to produce physiological stress responses, including increased secretion of cortisol and other stress-related hormones, disruption of circadian rhythms, and reductions in melatonin production. These effects are further exacerbated by inflammatory processes characterized by increased levels of C-reactive protein (CRP), interleukin-1 beta (IL-1β), interleukin-6 (IL-6), and tumor necrosis factor-alpha (TNF-α) [38,39,40]. Such systemic stress responses impair neural plasticity and cognitive performance, underscoring the effect of noise on cognition.

Nevertheless, the connection between physiological processes and the harmful effects of prolonged noise exposure remains to be thoroughly clarified. While the results of various studies are relatively consistent in demonstrating the adverse effects of noise on cognitive functions, the specific pathways involved remain ambiguous.

On the other hand, the impact effect on cognition could be attributed to the combination of other ambiental factors such as air pollution (e.g., particulate matter, carbon monoxide (CO), nitrogen dioxide) in urban environments, creating a “double burden” of environmental stressors that may independently or synergistically impair cognitive development through mechanisms such as neuroinflammation, oxidative stress, and blood–brain barrier disruption [41,42]. Moreover, socioeconomic disparities, including parental education, socioeconomic status (SES), and home environment quality, significantly moderate the neurocognitive impacts of noise exposure. Higher-SES households frequently attenuate noise-related risks through soundproof housing and cognitively enriched environments, which may buffer against developmental disruptions; in contrast, lower SES was associated with lower working memory ability in children [43,44]. Conversely, children from disadvantaged backgrounds are subjected to compounded risks, including chronic noise exposure, limited access to educational resources, and chaotic home environments that impede opportunities for sustained cognitive engagement [45].

The limitations of the reviewed studies primarily stem from their reliance on cross-sectional designs, which inherently limit the ability to identify causal relationships and fully explain the underlying mechanisms linking noise exposure to cognitive function. Additionally, various contextual factors were not consistently accounted for, such as the structural characteristics of educational facilities, proximity to industrial zones, and the potential compounding effects of air pollution or socioeconomic disparities. Failure to account for these factors risks overestimating noise-specific effects, as children exposed to both traffic noise and high particulate matter levels may experience compounded cognitive deficits not attributable to noise alone.

Individual exposure to noise prior to the study was not systematically measured, nor were participants’ subjective perceptions or sensitivities to noise, both of which could substantially mediate the observed effects. Furthermore, differences in cultural or linguistic contexts that might influence cognitive assessments were not addressed, which makes results difficult to compare between populations across studies.

Several studies also lacked adequate control for confounding variables, such as comorbid health conditions, sleep disturbances, or variations in teaching quality, which could independently affect cognitive outcomes. The small sample sizes in many studies further limit the generalization of their findings. Also, there was considerable variability in the cognitive assessment tools used, leading to challenges with inconclusive data.

Future research should aim to mitigate these limitations by incorporating longitudinal designs, larger and more representative cohorts, and robust control for potential confounders. Additionally, cognitive outcomes should be evaluated using culturally and linguistically validated neuropsychological assessments to enhance cross-study comparability. Moreover, studies should systematically control key confounders, including socioeconomic status, air pollution, sleep quality, and variations in educational environments, to better isolate the specific effects of noise exposure.

## 5. Conclusions

While this meta-analysis provides evidence that noise exposure may negatively impact cognitive function in children and adolescents, the findings should be interpreted with caution due to the high heterogeneity among studies and potential uncontrolled confounding factors. The variability in study designs, noise exposure assessments, and cognitive outcome measures limit the ability to draw definitive causal conclusions. Nonetheless, the observed effects underscore the importance of addressing environmental noise as a potential risk factor for cognitive development. Future research should prioritize longitudinal designs, standardized noise exposure assessments, and comprehensive cognitive evaluations while controlling key confounding factors.

## Figures and Tables

**Table 1:** Summary of keywords used in the search strategy.

Variables	Search Fields	Keyword
Independent	All Fields	Noise, acoustic, environmental noise, chronic noise, broadband, white noise
Dependent	All Fields	Cognition, cognitive, cognitive function, memory, learning, attention
Population	All Fields	Children, minor, youths, young, adolescents

**Table 2:** Summary of data extracted from research assessing noise exposure effects on cognitive function in youth.

Study	n1	m1	s1	n2	m2	s2	MD	SDp	SEp	d	se
Baek et al., 2023 [16]	480	105.9	15.57	509	109.0	16.30	−3.1	15.9499	0.5472	−0.2	0.0637
Tangermann et al., 2023 [3]	287	7.5	2.98	252	7.9	2.98	−0.4	2.9800	0.2573	−0.1	0.0915
Chere & Kirkham, 2021 [4]	43	132.85	176.67	43	285.72	285.42	−152.57	237.3572	51.1898	−0.6	0.2205
Connolly et al., 2019 [5]	335	6.7	3.2	334	7.4	4.3	−0.7	3.7893	0.2930	−0.2	0.0775
Bhang et al., 2018 [8]	134	110.04	1.29	134	116.54	1.11	−6.5	1.2034	0.1470	−5.4	0.1296
Seabi et al., 2012 [17]	151	30.16	13.76	191	40.95	14.06	−10.79	13.9284	1.5167	−0.8	0.1154
Söderlund et al., 2010 [18]	21	0.41	0.02	20	0.46	0.02	−0.05	0.0200	0.0062	−2.5	0.3323
Haines et al., 2001 [19]	236	37.11	1.02	215	38.97	1.03	−1.86	1.0248	0.0019	−1.8	0.1000

n1, number of participants of noise group; m1, noise mean; s1, noise SD; n2, number of participants of control group; m2, control mean; s2, control SD; MD, mean difference; SDp, pooled standard deviation; SEp, pooled standard error; d, SMD; se, standard error of SMD.

**Table 3:** Overview of study characteristics.

First Author	Year	Country	Sex (Male/Female)	Noise Source	Noise Value (dB)	Exposure Assessment	Place of Exposure	Age	Outcome	Measurement
Baek [16]	2023	Korea	520/469	Aircraft Noise	75 ≤ 80 dB	WECPNL	Schools	10–11	IQ	KIT-P
Tangermann [3]	2023	Switzerland	432/340	Home Road Traffic Noise	>55 dB	SiRENE project	Home	13–15	Memory	IST
Chere and Kirkham [4]	2021	UK	72/88	Environmental Noise	Perception of Noise	Questionnaire	Home	11–14	Executive function	Flanker (∆RT Accuracy score)
Connolly [5]	2019	UK	Not reported	Sound Events (chair, scrapes, pencil drops, and movement)	70 dB LAeq	HATS	Schools	11–16	Learning	The reading task (latency word learning)
Bhang [8]	2018	Korea	135/133	Road Traffic/ Aircraft Noise	60.8–62.8 dB	NS	Schools	10–12	IQ	KEDI-WISC
Seabi [17]	2012	South Africa	322/331 (181 did not respond)	Aircraft Noise	LAeq > 69–95 dBA	SVAN 955 Type 1	Schools	09–14	Learning	SRS2
Söderlund [18]	2010	Sweden	21/20	White Noise	78 dB	NS	Schools	11–12	Memory	Verbal episodic recall test
Haines [19]	2001	UK	229/222	Aircraft Noise	Leq > 63 dBA	NS	Schools	08–11	Memory and attention	Suffolk Reading Scale

WECPNL, weighted equivalent continuous perceived noise level; KIT-P, Korean Intelligence Test—Primary; IQ, intelligence quotient; L_Aeq_, equivalent average sound level A-weighted; HATS, B&K 4100 Head and Torso Simulator; NS, non-specificized, SRS2, The Suffolk Reading Scale Level 2; KEDI-WISC, Korean Wechsler Intelligence Scale for Children; IST, Intelligenz-Struktur-Test.

## Data Availability

All data supporting the findings of this study are included in the manuscript and its Supplementary Materials.

## Supplementary Materials

The following supporting information can be downloaded at https://www.mdpi.com/article/10.3390/neurosci6010022/s1, File Search S1: Comprehensive search strategy for articles to examine the effect of noise exposure on cognition abilities in children. File S2: Summary of studies excluded from the meta-analysis assessing the influence of noise exposure in the performance of cognition activities in children. File S3: R Code.
