# Supplementary material for: The Influence of Noise Exposure on Cognitive Function in Children and Adolescents: A Meta-Analysis
Source: NeuroSci. 2025 Mar 4;6(1):22. doi: 10.3390/neurosci6010022 (PMC11944768; doi:10.3390/neurosci6010022)
Supplement: Supplementary file 1 [file neurosci-06-00022-s001.zip › File S2.pdf]

S2. Summary of studies excluded from the meta-analysis assessing the influence of noise exposure in the performance of cognition activities in children.

Not Noise (2725)	Mental health conditions (64)	Not cognition (23)	Case report (107)	Not useful data (15)	Study Protocol (10)	Chapter/ Book/ Editorial/ comment (16)	Preprint (6)
R K Penney 1965	Göran B W Söderlund 2024	Tingting Yang 2024	Obuoma Umejuru Amaewhule 2024	Michelle Raess 2022	Erica Jostrup 2024	Rodrigo Pommot Berto 2024	Jose Inacio Costa-Filho 2024
J J Antonitis 1965	G Lelord 1973	B R Slater 1968	J L Schulma 1978	Maria Foraster 2022	Ilse E J I Coolen 2024	Giovanni Castellucci 2024	Chenguang Li 2024
A N Znamenskaia 1965	B S Reynolds 1974	L M Schell 1985	J L Edwards 1976	Ingrid Jarvis 2021	Elvira E Jimenez 2024	Maureen Hillier 2023	Wensi Wu 2024
J Schmiedeberg 1965	L Schreibman 1975	Marie Louise Bistrup 2006	H E Booker 1965	Elza Othman 2020	Kristoffer Vinther Olesen 2023	Kailey Remien 2023	Leon Ericsson 2023
J D MCNEIL 1965	R L Koegel 1976	M Bhardwaj 2013	J Hogg 1983	Charlotte Clark 2012	Ismaël Atchia 2021	Jennifer K Whiting 2022	Abigail Casalvera 2023
R S WILSON 1964	Chantal Kemner 2002	Chen-Yin Tung 2013	C Johnston 1981	Elise vanKempen 2011	Roberta W Scherer 2014	QSM Challenge 2.0 Organization Committee 2021	Kang Jin 2023
G M FACCHINI 1964	Jury D Kropotov 2005	E Bocaege 2016	A P Schneider 2nd 1983	Mark Matheson 2010	Friedemann Geiger 2013	André Schultz 2019	
P S SHPAKOV 1964	M E Toplak 2005	Halide Çetin Kara 2023	S Q Cohlan 1984	Stephen Stansfeld 2010	Laura E Ellington 2012	Avraham Beigelman 2018	
S H LOVIBOND 1963	Göran Söderlund 2007	Jun Wang 2024	J L Emery 1984	Patricia J Bauer 2010	Emma Sciberras 2010	Kathleen Rastle 2018	
M WERTHEIMER 1961	Michelle A Shanahan 2008	You-Rak Choi 2024	C M Temple 1984	Godson R E E Ana 2009	Monique Delaroche 2004	Shimin Fu 2018	
E Maltzman 1965	Stuart J Johnstone 2007	Wenyan Qiao 2024	R G Weleber 1983	Bridget M Shield 2007		John E Opfer 2011	
H Lobb 1960	Carole Tardif 2007	Youness Arjoune 2024	W W Baber 1988	Charlotte Clark 2006		S A Stansfeld 2005	
L Michaux 1966	Daniel Lenz 2008	Daniesha S Hunter-Rue 2024	M Kattan 1985	S A Stansfeld 2005		Paul Bloom 2004	
N K Glukhova 1967	Rhea Paul 2008	Eleanor R Palser 2024	J M Aiken 1984	Matsui 2004		P Tallal 2000	
L Schoer 1967	R Matt Alderson 2008	Teddy Zhao 2024	M M Logvinoff 1990	T R Fenton 1974		M R Evans 1996	
D Guess 1967	Francisco J Sanchez-Marin 2008	Avraham Beigelman 2024	M Rizzo 1989			J G Ayres 1996	
R I Polikanina 1967	Andrew Cook 2014	Xianxian Li 2024	C M Temple 1985				
S Fraiberg 1966	Jennifer A Mautone 2012	Zeng Zhang 2024	D Niedecken 1991				
E S Ohlrich 1968	Maria Casagrande 2012	Lihi Sarfaty 2023	H M Yerman 1990				
Y Brackbill 1968	Suzannah K Helps 2011	Chiara Visentin 2023	T Koeda 1992				
G Karsdorf 1968	Susan O Latham 2014	Mariangela Lopes Bitar 2018	L J Mazur1995				
G S Liakh 1968	Hung-Yu Lin 2017	Neha Thakur 2016	F Bernardi 1995				
R I Polikanina 1969	Simon Baijot 2016	Nikolaos S Chatzakis 2014	I A Hyman1998				
J A Cheyne 1969	Emily Neuhaus 2015		H Melhus 1998				
K Connolly 1969	Karin Vanessa Carias 2019		O Iu Karpova 1996				
W J Meyers 1969	Julie E Cubicciotti 2019		K Shamboul 1999				
D J Bakker 1970	Marleen F Westerveld 2018		R Caraballo 2000				
L G Voronin 1970	Songpoom Benyakorn 2018		A Pisanti 2000				
J R Haynes 1970	Maik Pertermann 2019		D K Ng 2000				
P S Massey 1969	Sabrina Turker 2019		Wendy A Stewart 2002				
D P Wyon 1970	Shulamite A Green 2019		M Bitsori 2001				
A N Nagy 1970	Ruimin Wang 2019		Robert J Freishtat 2002				
A J Sameroff 1970	Yunkai Zhang 2021		M W Sung 2001				
L S Schoenfeld 1970	Majid Moghaddari 2020		T Hilliard 2003				
D J Bakker 1970	R R Wildeboer 2020		M A Kurian 2003				
Z V Beliaeva 1970	Suraj Singh Senjam 2021		D T W Teo 2004				
T Moreau 1970	Kana Ram Jat 2020		Tonny Mampilly 2005				
J E Favell 1972	Xiaoying Pan 2020		Emilia María Hidalgo Castro 2004				
I S Serikov 1972	Ying Chen 2020		Scott D Buckley 2006				
N McConaghy 1972	I-Chen Chen 2022		Alessandro Mantovani 2007				
N J Cohen 1972	Yibin Tang 2022		John T Rapp 2007				
C Tomlinson-Keasey 1972	Amanda Nally 2021		Alice-Ann Darrow 2007				
R Clifton 1972	Wayne J Wilson 2021		Scott E Brietzke 2007				
A Silverstein 1972	Hongyi Zhao 2021		S Marinho 2007				
B Bartholomeus 1971	Brennan P Terhune-Cotter 2021		Michalle Soudack 2007				
R C Katz 1971	Xiang-Yun Liu 2021		Simon J Davis 2007				
J S Birnbrauer 1971	Itai Berger 2021		Christine L Allison 2007				
O I Lovaas 1971	Jihye Ryu 2022		Deepti Suri 2008				
L S Elliott Jr 1971	Sandra García-Ponsoda 2024		Kerstin H Kipp 2008				
M Lewis 1971	Hong Zhang 2024		Lokesh Tiwari 2009				
T A Crul 1971	Erica Jostrup 2024		Christophe Chardot 2009				
J A Cheyne 1971	Charlotte Dumont 2024		Susan S Johnston 2009				
E A Holden Jr 1971	George Gifford 2024		Bungo Nishimura 2008				
W H Miller 1971	Mohammad Reza Yousefi 2024		S Caimmi 2011				
M Berel 1971	Jian Zhang 2024		Takahiro Yonekawa 2011				
J H Hollis 1971	Maria Luongo 2024		Emma Matthews 2011				
V V Aliakrinskiĭ 1971	Vahid Nejati 2024		Thomas G Sternberg 2010				
W D Fenz 1971	Yu Chen 2023		Hans Balink 2012				
J Fassler 1970	Erica Jostrup 2023		J D Binham 2012				
T D Borkovec 1970	Cristina Ardura-Garcia 2023		Sunit C Singhi 2011				
N Accornero 1970	Orhan Atila 2023		L F A D Moura 2012				
M Powazek 1973	Pengbo Wei 2023		Hans Balink				
M Kinnealey 1973	Martin H Maurer 2022		Tara Olivia Loughrey 2014				
G Grünewald 1973	Florence Véronneau-Veilleux 2022		Kara L Wunderlich 2014				
K N Mcnicol 1973			Yeamie M Kousari 2014				
P Tallal 1973			Sylvia Klinkenberg 2014				
R W Collins 1973			Rachel Scalzo 2015				
D A Siddle 1973			Courtney T Chou 2016				
G C Young 1973			Mara Lelii 2016				
G Lelord 1973			Frederick J Menick 2016				
D H Marsh 1973			Marianna Alesi 2016				
E Carrow 1973			Francesca Tedoldi 2016				
D J Duché 1973			Giorgio Cozzi 2018				
J E Hasazi 1973			Kara L Wunderlich 2017				
B C Carruthers 1973			James Paton 2017				
R J Simensen 1973			Renu Khetan 2018				
R Forehand 1973			Theodora Nelson 2017				
F Laffont 1973			Carolyn Goldberg Butler 2018				
D T Siddle 1973			Mohamed Elyadari 2017				
O Bernath 1973			Ali Yurtseven 2017				
W E Stephens 1973			Kiley A Schneider 2018				
W S Millar 1972			José Ferreira Penêda 2019				
H Kassinove 1972			Tracy L Kettering 2018				
J Melnick 1972			Ana Luiza Roncati 2019				
D A Siddle 1972			Matthew R Purkey 2019				
R E Fleer 1972			P Reynard 2020				
W K Berg 1972			Elena Chiappini 2019				
D Werden 1972			Seana Molloy 2021				
E F Boyd 1974			Francesca Corrias 2021				
R E Culp 1974			Priyanka Thakeria 2019				
L Y Paden 1974			A Tsagkovits 2020				
J Kagan 1974			Nilay BaŞ İkİzoĞlu 2019				
R B McCall 1974			Jill C Fodstad 2021				
F D Horowitz 1974			Farah Salman Alnemari 2024				
P Combe 1974			Jashpal Yadav 2024				
S B Stainback 1974			Heidi Makrinioti 2024				
W S Millar 1974			María Suárez-Bustamante Huélamo 2024				
C Spring 1974			Sher Singh Dariya 2023				
T Moreau 1974			Álvaro Torres-Martos 2023				
M Sidman 1974			Leonardo M F De Souza 2021				
T J Berndt 1974			Qu-Ming Zhao 2021				
R K Clifton 1974			Hiromi Teranishi 2021				
H S Frank 1974			A Molina 2021				
B Butler 1974			Munanura Turyasiima 2021				
R M Alworth 1974			Haruo Fujino 2020				
P Pothier 1974			Mitsuro Chiba 2020				
T J Mulhern 1974			Maurizio Barbara 2022				
A Silverstein 1974							
W K Berg 1974							
R Meares 1974							
R B McCall 1974							
E L Glynn 1974							
R Hirschman 1974							
G Gutezeit 1974							
G C Young 1973							
S S Kupietz 1976							
D K Kinney 1976							
M Dixon 1976							
J Boucher 1976							
R Elliott 1976							
L A Streeter 1976							
G Lelord 1976							
H Kotses 1976							
R J Call 1975							
J E Hasazi 1975							
D H Warren 1975							
J F Williams 1975							
J K Whittaker 1975							
A A Baumeister 1975							
S J Hutt 1975							
B Kaltsounis 1975							
S Millar 1975							
S Millar 1975							
K Bloom 1975							
D S Geffner 1975							
S J Wormith 1975							
M Perlmutter 1975							
R D Tweney 1975							
E R Browning 1974							
K W Laub 1974							
A Morton-Evans 1978							
C L Denton 1978							
L F Salzman 1978							
E A Baum 1978							
R J Pruhs 1978							
A Kankkunen 1978							
H W Chang 1977							
R E Remington 1977							
A Rincover 1977							
E A Holden 1977							
L P Lipsitt 1977							
J R Thomas 1977							
W C Chasey 1977							

W W Finley 1977<br>M Dauzat 1976<br>J M Kraegel 1976<br>C S Watson 1976<br>G E Taylor Jr 1976<br>E Kurth 1976<br>D Carroll 1976<br>L Ricketts 1976<br>A Leviton 1976<br>E Klotzbücher 1976<br>H M Doan 1976<br>P Poorman 1976<br>J Martineau 1980<br>R D Bailey 1980<br>L L Elliott 1980<br>J F Camus 1980<br>O Fleiss 1980<br>G Poulsen 1980<br>M J Mendelson 1979<br>D Muir 1979<br>R B Monsen 1979<br>R Cass 1979<br>J Hogg 1979<br>J L Schulman 1979<br>M E Horn 1979<br>I B Perelle 1979<br>P R Brooks 1979<br>B Novick 1979<br>J Goldberg 1979<br>R F Jarman 1979<br>C K Whalen 1979<br>M D Vernon 1979<br>J Gould 1979<br>R Cammann 1979<br>L A Streeter 1978<br>F Frankel 1978<br>G W Hynd 1978<br>J E Patton 1978<br>E A Baum 1978<br>J Haskett 1978<br>E B Lehman 1982<br>S D Marcus 1982<br>D K Mitra 1981<br>G S Grosser 1981<br>A M Richardson 1981<br>T J Fria 1981<br>C D Bluestone 1981<br>M L Serafine 1981<br>M L Smith 1981<br>E Pollitt 1981<br>L E Bahrick 1981<br>M T Ivancic 1981<br>G S Liptak 1981<br>D W Johnson 1981<br>R A Prentky 1981<br>G Lamberti 1981<br>T Kuberski 1981<br>J M Hasbrouck 1980<br>D J Kolko 1980<br>C Djerassi 1980<br>F C Bennett 1980<br>J S O'Shea 1980<br>S Graham 1980<br>M J O'Connor 1980<br>S M Davis 1980<br>R S Bundy 1980<br>B Crassini 1980<br>M S Knight 1983<br>J C McDermott 1983<br>F A Saunders 1983<br>D A Lee 1983<br>A N Speight 1983<br>T E Liston 1983<br>A Jütte 1983<br>V M Howie 1983<br>C D Bluestone 1983<br>M Tos 1983<br>N N Singh 1983<br>J E Obrzut 1983<br>D G Tinkelman 1983<br>M E Martin 1982<br>P J Helders 1982<br>L F Lowenstein 1982<br>E Goodall 1982<br>M J Mendelson 1982<br>T I Hatae 1982<br>R C Beckerman 1982<br>S Friel-Patti1982<br>P R Jones 1982<br>C H Sorensen 1982<br>K Parker 1985<br>O Elbrønd 1985<br>J L Koltai 1984<br>B Magnuson 1984<br>J Martineau 1984<br>V F Konovalov 1984<br>S Steven 1984<br>R K Clifton 1984<br>H van Engeland 1984<br>P H Miller 1984<br>A H Little 1984<br>V I Correa 1984<br>J Y Mok 1984<br>S Peeke 1984<br>A Bylander 1984<br>L Kay 1984<br>P Narcy 1984<br>A Okwesa 1983<br>P Dermody 1983<br>K A Hansen 1983<br>F Alvarez 1983<br>R J Zatorre 1983<br>J C Gershel 1983<br>S R West 1983<br>S L Blackwell 1983<br>A A Ibatullina 1983<br>E S Park 1986<br>H R Anderson 1986<br>A G Gordon 1986<br>M T Wheeler 1986<br>G Murphy 1986<br>M L Batshaw 1986<br>M Fiellau-Nikolajsen 1986<br>R C Welliver 1986<br>P F Wright 1985<br>P Bonding 1985<br>P Dellenbach 1985<br>R Rogozea 1985<br>B T Cooper 1985<br>M Tos1985<br>W G Thomas 1985<br>A S Walker-Andrews 1985<br>M Tos 1985<br>D E Comings 1985<br>M L Casselbrant 1985<br>R S Fischler 1985<br>D P Strachan 1985<br>M A McHenry 1985<br>G Bartolozzi 1985<br>L Birch 1985<br>G Póder 1985<br>G S Waters 1988<br>J Lous 1988<br>R L Greene 1988<br>D L Fisher 1988<br>M H Charlop 1988<br>M M Marcell 1988<br>D E Smith 1988<br>P A Szeszulski 1987<br>J Martineau 1987<br>M F Guill 1987<br>B A Morrongiello 1987<br>G Nigro 1987<br>R S Johnston 1987<br>R P Mills 1987<br>H Lee 1987<br>M Bax 1987<br>F R Manis 1987<br>D P Skoner 1987<br>L Birch 1987<br>E J Duiverman 1987<br>K Toyoshima 1987<br>K D Allen 1987<br>E F Cserháti 1986<br>D N Brooks 1986<br>J S O'Shea 1986<br>T Jacob 1986<br>M C Allen 1986<br>M de Montfort Supple 1986<br>R A Hill 1989<br>J Friend 1989<br>D M Stack 1989<br>M S Victoria 1989<br>Z Koszarny 1989<br>B M Lester 1989<br>M J Weiss 1988<br>N N Singh 1988							

P Fürst 1988<br>R Bermejo 1988<br>M Mior 1988<br>E Rylander 1988<br>P B McCray Jr 1988<br>W von Suchodoletz 1988<br>D W Haffner 1988<br>E Marosi 1990<br>K D Allen 1988<br>M M Awad 1991<br>C T Le 1991<br>J Mertsola 1991<br>H Møller 1990<br>G S Goodman 1990<br>E E Perris 1990<br>C O Oyejide 1990<br>K A Waldron 1990<br>P J Thompson 1990<br>K Wynn 1990<br>Z Mailloux 1990<br>E Marosi 1990<br>L I Peresleni 1990<br>L I Katin 1990<br>W G Masten 1989<br>M E Pichichero 1989<br>E M Mandel 1989<br>J L Gillmore 1989<br>H Pratt 1989<br>J K Torgesen 1989<br>J Schäfer 1989<br>J L Ballvé Moreno 1989<br>S L Voelker 1989<br>M Shohat 1989<br>F W Henderson 1992<br>S Siegel 1992<br>S E Stangerup 1992<br>S Fudge 1992<br>L Bentur 1992<br>G Wennergren 1992<br>J S Gravel 1992<br>G Póder 1992<br>O M Chay 1992<br>W E Merriman 1991<br>D H Ashmead 1991<br>E Tirosh 1991<br>B A Morrongiello 1991<br>Y Z Lin 1991<br>S Halken 1991<br>H Letchman 1991<br>G A Zielhuis 1991<br>G L Strope 1991<br>D K Walker 1991<br>D A Pearson 1991<br>V Benigno 1991<br>C Stenström 1991<br>A D Genazzani 1991<br>A L Wright 1991<br>N A Badian 1991<br>M Baldo 1993<br>M J Young 1993<br>J L Huang 1993<br>R H Felton 1993<br>D L Duffy 1993<br>R Lipkens 1993<br>M S Atkins 1993<br>I Johansson 1993<br>J M Samet 1993<br>R H Kraft 1993<br>H E Lemoine 1993<br>D C Persellin 1993<br>G Cossu 1993<br>I Varsano 1993<br>S Hygge 1993<br>B Johansson 1992<br>K Veit 1992<br>D J Lewkowicz 1992<br>C M Mellis 1992<br>E Service 1992<br>J Martineau 1992<br>H J Steen 1992<br>C A Mangina 1992<br>T K Ninan 1992<br>M Baldwin 1992<br>L E Bahrick 1992<br>L Hillier 1992<br>H M Chaĭchenko 1992<br>M S Siniaia 1995<br>S Arehole 1995<br>J S Gravel 1995<br>F D Martinez 1995<br>B K Dolenko 1995<br>H J Singh 1995<br>T Rinne 1995<br>A Høst 1994<br>M A Epstein 1994<br>H P Sachdev 1994<br>S Sugama 1994<br>D K Luyt 1994<br>J B Clough 1994<br>J M Silvestri 1994<br>L Kuikka 1994<br>J Deerojanawong 1994<br>K N Desager 1994<br>B F Fuller 1994<br>D T Jones 1994<br>H F Chitiri 1994<br>B Culpepper 1994<br>V H van Daal 1994<br>I M Balfour-Lynn 1994<br>J Q Claessen 1994<br>E Fox 1994<br>J H Satterfield 1994<br>A M Brooke 1996<br>N Meiran 1996<br>V J Carey 1996<br>S A Zeisel 1995<br>R D Krugman 1995<br>L H Harrison 1995<br>N J Freezer 1995<br>R J Croswell 1995<br>M Stuart 1995<br>D J Majsterek 1995<br>A L Wright 1995<br>M G Clarkson 1995<br>K C Lødrup Carlsen 1995<br>C A Silverman 1995<br>J M Martìnez-Selva 1995<br>F W Henderson 1995<br>G E Lancioni 1995<br>F D Martinez 1995<br>J B Clough 1995<br>K H Carlsen 1995<br>A Chhabra 1995<br>N Wilson 1995<br>C H Kennedy 1995<br>R H Kraft 1995<br>S C Hogan 1997<br>J E Klig 1997<br>T Heiskanen-Kosma 1997<br>R A Cureton-Lane 1997<br>M P Robb 1997<br>J D Vince 1996<br>E M Mandel 1996<br>Y Takahashi 1996<br>C V Powell 1996<br>R Seidl 1996<br>C M Walsh-Kelly 1996<br>S H Kelder 1996<br>E M Ornitz 1996<br>J M Annett 1996<br>G Bentham 1996<br>N L Gotteiner 1996<br>M Gleeson 1996<br>D W Dockery 1996<br>M J Hanson 1996<br>V L Bianki 1996<br>T Reijonen 1996<br>C Hirano 1996<br>A L Wright 1996<br>R Buchdahl 1996<br>S C Hogan 1996<br>H Roizin 1996<br>L L Hunter 1996<br>M Samuel 1998<br>J R Villa 1998<br>P Bryant 1998<br>B Volovitz 1998<br>J M Court 1998<br>P J Gergen 1998<br>A Breborowicz 1998<br>H Martinez 1998<br>A Seidler 1998<br>S Vanden Ark 1997<br>E M Ornitz 1997<br>G L Christie 1997<br>M A Baeza Bacab 1997<br>S E Gathercole 1997							

I J Doull 1997<br>B V Ustiushin 1997<br>C H Song 1997<br>S Shimojo 1997<br>T M Reijonen 1997<br>D Y Koller 1997<br>S S Rothenberg 1997<br>L G Duncan 1997<br>M E Lavoie 1997<br>E Bialystok 1997<br>C Dezateux 1997<br>N M Wilson 1997<br>S M van Schaik 1999<br>G F Fox 1999<br>J Taylor 1999<br>C S Ho 1999<br>M Cheour 1999<br>K I Yau 1999<br>C Dezateux 1999<br>S F Stokes 1999<br>T W Powell 1999<br>J Sánchez 1999<br>S Millar 1999<br>J Brouard 1999<br>N Bowles 1998<br>O Karaman 1998<br>F D Martinez 1998<br>K Oymar 1998<br>S A Moody 1998<br>K O Rivers 1998<br>U Staden 1998<br>C Hulme 1998<br>M Samuel 1998<br>R Treiman 1998<br>T M Reijonen 1998<br>D Baker 1998<br>J Spee-van der Wekke 1998<br>G Lis 1998<br>F De Baets 1998<br>V W Berninger 2000<br>L A Henry 2000<br>S C Leech 2000<br>T Koeda 2000<br>O S Levine 1999<br>E Ferekidis 1999<br>V W Berninger 1999<br>R Ceponiene 1999<br>P H Seymour 1999<br>P K Pattemore 1999<br>A Blanco-Quirós 1999<br>R A Pretlow 1999<br>K C Mittag 1999<br>R Kilpeläinen 1999<br>F H Duffy 1999<br>S Vlachou 1999<br>I Orbach 1999<br>R Kilpeläinen 1999<br>H T Chang 1999<br>R H Fishbein 1999<br>D Pavón 1999<br>E M Ornitz 1999<br>B L Matthews 1999<br>O Karaman 1999<br>W J Rodriguez 1999<br>J Henderson 1999<br>F Alba Moreno 1999<br>T M Reijonen 2000<br>L G Duncan 2000<br>D C Fowles 2000<br>A D Baxter-Jones 2000<br>R G Najm-Briscoe 2000<br>S Gissel 2000<br>J Boucher 2000<br>Y S Sininger 2000<br>D Annequin 2000<br>T M Ball 2000<br>J Champion 2000<br>M Moore 2000<br>M Eriksson 2000<br>P F de Jong 2000<br>O Hetlevik 2000<br>G Dutau 2000<br>R H Margolis 2000<br>L Kay 2000<br>V M Zhenilo 2000<br>L Bont 2000<br>J F Rovet 2000<br>C M Fletcher-Flinn 2000<br>R Buchdahl 2000<br>S Rietveld 2000<br>J Oakhill 2000<br>F Oğuz 2000<br>U Pajari 2001<br>J Sunyer 2001<br>R Kahana-Kalman 2001<br>L Bont 2001<br>F Buchvald 2001<br>J A Castro-Rodríguez 2001<br>A L Wright 2001<br>S Illi 2001<br>D Skarlatos 2001<br>M T Stein 2001<br>V Grech 2001<br>J H Park 2001<br>D L Young 2001<br>S Chady 2001<br>I Tarnanas 2001<br>N A Fox 2001<br>S Esposito 2000<br>J Day 2000<br>M R Cardoso 2000<br>Y Nagayama 2001<br>H C Lin 2001<br>M Dennis 2001<br>C Calvo Rey 2001<br>V Gumenyuk 2001<br>L Tano 2001<br>S Molholm 2001<br>M M Haines 2001<br>R G Ehrenberg 2001<br>C Delacourt 2001<br>K Spencer 2001<br>J A Calhoon 2001<br>J A Calhoon 2001<br>J R Saffran 2001<br>B Arnoux 2001<br>K Oymar 2001<br>H P Sachdev 2001<br>D Loke 2001<br>I Valencia 2001<br>F Rusconi 2001<br>S Y Bhave 2001<br>I Azevedo 2001<br>MRC Multi-centre Otitis Media Study Group 2001<br>R A Mevorach 2001<br>Young Yull Koh 2002<br>Julie Wakefield 2002<br>Lorraine E Bahrick 2002<br>Th D Mavrommati 2002<br>Philip Seliger 2002<br>Jung-Chang Tang 2002<br>Marjorie Gang 2002<br>Panagiotis G Simos 2002<br>A-L Ponsonby 2002<br>Juan C Celedón 2002<br>Christine M Walsh-Kelly 2002<br>G Cirrincione 2002<br>Hartmut Ising 2002<br>Janet E Farmer 2002<br>Francis Trépardoux 2002<br>Glen P Aylward 2002<br>Andréa L Guimarães Teldeschi 2002<br>Larry W Hawk Jr 2003<br>Mari Miyoshi 2002<br>Anne Kotaniemi-Syrjänen 2002<br>Juan C Celedón 2002<br>Tapanok Rattanadilok Na Bhuket 2002<br>Jaak Panksepp 2002<br>Conor Liston 2002<br>Xiao-Mei Mai 2002<br>Laurie S Eisenberg 2002<br>Mirna M Farah 2002<br>Peter P Van Asperen 2002<br>Louis I Landau 2002<br>Stephanie Moody Antonio 2002<br>P Pala 2002<br>B Young 2002<br>Susanna Esposito 2002<br>Thierry Nazzi 2002<br>Yutaka Takahashi 2002<br>G Corvo 2003<br>S Lau 2003<br>M Silverman 2003<br>M Giovannini 2003<br>Laurens P Koopman 2003<br>Andrea Facoetti 2003<br>Aimee L Campbell 2003							

R E Sapien 2003							
Anne Kotaniemi-Syrjänen 2003							
Gregg B Nelson 2003							
Patricia C Chulada 2003							
M Aoyagi 2003							
Chorng-Yann Su 2003							
Judith A Thorne 2003							
C Bez 2003							
Barbara A Morrongiello 2003							
Anne Kotaniemi-Syrjänen 2003							
Anne-Louise Ponsonby 2003							
Johannes C Ziegler 2003							
Carmen M Ruiz-Jarabo 2003							
X M Mai 2003							
Lorena Cifuentes 2003							
Benjamin Volovitz 2003							
Gary W Evans 2003							
Jennifer S Lipton 2003							
Anna Shestakova 2003							
E H Aylward 2003							
Mircea Nanulescu 2003							
Hsiang-Ju Huang 2003							
T Fernández 2003							
Isabelle Meyts 2003							
Roger P Weissberg 2003							
Sarah C M Hogan 2003							
Milton J Schmida 2003							
Tobias Obst 2004							
David G Thomas 2004							
N Katier 2004							
John Woodward 2004							
Claire E Wakefield 2004							
Leigh Ann Peteani 2004							
Valentina Gumenyuk 2004							
Aneta Dimoska 2003							
A Cano Garcinuño 2003							
Noreen M Clark 2003							
Douglas L Roy 2003							
Jung-Chang Tang 2003							
Vijay Kandala 2003							
Abraham Oommen 2003							
David H Newman 2003							
F H Santos 2003							
Paul Shaw 2004							
James E Connell 2004							
Gregory Redding 2004							
Tricia Striano 2004							
Chuen-Der Kao 2004							
T Lepistö 2004							
Margaretha L Casselbrant 2004							
Rikki Mistry 2004							
A Le Louarn 2004							
Kathrin Negele 2004							
Rena M Eenshuistra 2004							
Patricia G Stelmachowicz 2004							
Alejandro M Teper 2004							
Nora Al-Nahedh 2004							
Małgorzata Steczkowska-Klucznik 2004							
V A Bronnikov 2004							
G Emenius 2004							
Catherine Karr 2004							
Eli Shahar							
B Tokar 2004							
David L Share 2004							
Stefano Guerra 2004							
Peter F de Jong 2004							
Eli Vakil 2004							
Ozkaya Emin 2004							
Lorraine E Bahrick 2004							
L Bont 2004							
Ruth Soferman 2004							
D Berwanger 2004							
T Goetghebuer 2004							
Louis Bont 2004							
Noel L Cohen 2004							
Anne-Louise Ponsonby 2004							
Anne-Louise Ponsonby 2004							
Murray J Dyck 2004							
Barbara Velsor-Friedrich 2004							
Andrea Facoetti 2005							
E Mark Mahone 2005							
Janghan Lee 2004							
Ching-Wen Liou 2004							
Jenny Philip 2004							
Shun Sagai 2004							
Gerri Hanten 2004							
D Mak 2004							
David B Pisoni 2004							
D Erik Everhart 2004							
T Matsui 2004							
Lea Bentur 2004							
Fabio Midulla 2004							
Stephan Bender 2004							
Predrag Radivojac 2004							
Stephan Bender 2004							
Jing Huang 2004							
Maria Helena D Benício 2004							
Margaret Harris 2004							
Fabienne Collette 2005							
Beverly J Trezek 2005							
Jennifer Simpson 2005							
Jinho Yu 2005							
Neşe Celebisoy 2005							
S Hélène Deacon 2005							
Andrés Heerlein L 2005							
Bernt Alm 2005							
V Jayarajan 2005							
A A Aleksandrov 2005							
Hoda M Malaty 2005							
Dale Borman Fink 2005							
Ana Paula Corona 2005							
Thatiana Del Carlo Furbeta 2005							
Karen Banai 2005							
David Skuse 2005							
Teresa McNally 2005							
Katrin Stumptner 2005							
H Bilolikar 2005							
Deborah L Speece 2005							
Moema N Chatkin 2005							
Young J Juhn 2005							
Rim H Al-Samsam 2005							
Roger W Li 2005							
Sara Määttä 2005							
Hans-Olav Fjaerli 2005							
Christian Hermann 2005							
V Yu Zaitsev 2005							
Gary W Evans 2005							
Eugenia Costa-Giomi 2005							
M Sznajder 2005							
E T Kandelaki 2005							
Brendan A Rich 2005							
Anne Kotaniemi-Syrjänen 2005							
Dakshesh Parikh 2005							
Astrid Geudens 2005							
John Xenellis 2005							
Charalabos Papageorgiou 2005							
Ayse Tana Aslan 2005							
J A Castro-Rodríguez 2005							
Panagiotis G Simos 2005							
Heather J Wassall 2005							
Duane L Sherrill 2005							
Hawley E Montgomery-Downs 2006							
Marieke Zwaanswijk 2006							
Emilio Escuer Acín 2006							
Iris Levin 2006							
E J Kay 2006							
Timothy R Huerta 2006							
Dominic A Fitzgerald 2006							
C Spironelli 2006							
Sharon Vaughn 2006							
Joshua K Hartshorne 2006							
Elaine R Silliman 2006							
Kenn Apel 2006							
Allan Linneberg 2006							
James E Gern 2006							
Clare S Murray 2006							
Blanca Morfín-Maciel 2006							
Feiyan Chen 2006							
Fabrizio Drago 2006							
Amy S Desroches 2006							
A Liasis 2006							
Benoît Jutras 2006							
Anita L Kozyrskyj 2006							
Tuomas Jartti 2006							
Peter J Hatcher 2006							
Hans Bisgaard 2006							
Theresa W Guilbert 2006							
Sarwer Jameel Siddiqui 2006							
Mary Smale 2006							
Jaime Sánchez 2006							
Markus Lilja 2006							
Joanna Gasiorowska 2006							
Arielle Borovsky 2006							
Sammy Perone 2006							
Roberto Anaya-Prado 2006							
Irene Sideris 2006							
David E Kuhn 2006							

Peter van den Hazel 2006<br>Christian Hermann 2006<br>Takako Fujioka 2006<br>Lisa C Liberman 2006<br>Jutta Kray 2006<br>Matthias R Mehl 2006<br>E S Chauliac 2006<br>Olga Nacajauskaite 2006<br>Carmel Houston-Price 2006<br>Wiesław Jeedrychowski 2007<br>D R Watson 2007<br>Clare D Ramsey 2007<br>Stuart J Johnstone 2007<br>Jason L Anthony 2006<br>Eliara Pinto Vieira 2007<br>Min-Sup Shin 2007<br>Nerin N Bahceciler 2007<br>Hasan Yuksel 2007<br>Kazim Karaaslan 2007<br>Leonard B Bacharier 2007<br>Sandra E Trehub 2007<br>Pasi Lehtinen 2007<br>Robert B Belshe 2007<br>Joanna Gasiorowska 2007<br>Przemko Kwinta 2007<br>Jenny Ploeg 2007<br>Jenny Ploeg 2007<br>Alexandra Carmen Câra 2007<br>Gerri Hanten 2007<br>Kuender D Yang 2007<br>Debra A Stern 2007<br>Saskia de Graaff 2007<br>Olufemi Jaiyesimi 2007<br>Katharina Manassis 2007<br>Robert Pasnak 2007<br>Susanne Grassmann 2007<br>Mary D Klinnert 2007<br>Howard M Corneli 2007<br>Herberto J Chong Neto 2007<br>Hsin-Jen Tsai 2007<br>Tuomas Jartti 2007<br>Javier Mallol 2007<br>Chao He 2007<br>B Crishna 2007<br>Alfredo E Hoyos 2007<br>Allyson Hewitt 2007<br>Man Tian 2007<br>N Cortés Alvarez 2007<br>Jackie L Micklewright 2007<br>Michael C Stevens 2007<br>Roger W Li 2007<br>Peter F de Jong 2007<br>Yung-Yang Lin 2007<br>Tongran Liu 2007<br>Zhuang-Gui Chen 2007<br>Michael P Robb 2007<br>Thomas J Baker 2008<br>Christina M Krause 2008<br>Erin Horrocks 2008<br>Bouwien C M Smits-Engelsman 2008<br>Rajesh Kumar 2008<br>Claudine Bowyer-Crane 2008<br>Elisabeth Glass 2008<br>Luis J Goldman 2008<br>Chad W Tiernan 2008<br>Linda M Gonzalez 2008<br>Graeme Fairchild 2008<br>Kimberly Horn 2008<br>Feilin Hsiao 2008<br>Daphne S Cain 2008<br>Günter Krampen 2008<br>Miguel Luiz de Sant Ana Angeli 2008<br>Suzanne Schuh 2008<br>Plamenka Nanova 2008<br>P M Matricardi 2008<br>Sharon E Watt 2008<br>Ian Smythe 2008<br>Elisabeth Glass 2008<br>Rosângela Melo Vasconcelos 2008<br>S Bosis 2008<br>Zoheir El-Hoshy 2008<br>Michael Hoa 2008<br>Elizabeth Nilsen 2008<br>William J Doyle 2008<br>David L Neumann 2008<br>Lisa Noble Weiss 2008<br>Bernt Alm 2008<br>C Kiese-Himmel 2008<br>Sebastian Jentschke 2008<br>Nevin Uzuner 2008<br>Desmond Sergeant 2008<br>Mina C Johnson-Glenberg 2008<br>Juliana Nunes Santos 2008<br>Lionel G Standing 2008<br>Robert S Tepper 2008<br>William E Merriman 2008<br>Daniel J Jackson 2008<br>Mandy S Plumb 2008<br>Sammy Perone 2008<br>H Julia Hannay 2008<br>Svein Eikeseth 2009<br>Patricia Bauer 2009<br>Przemko Kwinta 2009<br>Craig M Wright 2009<br>Bradley R King 2009<br>Zoe L Quick 2009<br>Diego G Peroni 2009<br>C J Llapur 2009<br>R Geva 2008<br>Eleanor D Brown 2008<br>Leonard B Bacharier 2008<br>David L Neumann 2008<br>Jacob Urkin 2008<br>Anja C Lepach 2008<br>Juliana Roda 2008<br>Jeanne L Shinskey 2008<br>Marie S Burrage 2008<br>Marialuisa Martelli 2009<br>Marieke van Heugten 2009<br>Jennifer J Vannest 2009<br>David E Wolfe 2009<br>Javier Mallol 2009<br>Herberto José Chong Neto 2009<br>Noreen M Clark 2009<br>L M Borrego 2009<br>Michelle R Ellefson 2009<br>Eric Winer 2009<br>Gonzalo Valdivia C 2009<br>Judy Plantinga 2009<br>Hsin-Jen Tsai 2009<br>Donald F Sacco 2009<br>Michelle G Craske 2009<br>Geórgia Chalfun 2009<br>Kevin Marks 2009<br>Joel Fluss 2009<br>Allison M Waters 2009<br>Rachel Barr 2009<br>Manuel García-Magariños 2009<br>Wenjung Wang 2009<br>Leonard B Bacharier 2009<br>Lei Wang 2009<br>Malgorzata E Wilinska 2009<br>Marc J Lanovaz 2009<br>Nasar Asl Aminabadi 2009<br>R Malatesha Joshi 2009<br>Inés de Mir Messa 2009<br>B Vachha 2009<br>Anne Castles 2009<br>Carlos Diaz-Vazquez 2009<br>Per Lagerløv 2009<br>Kathie Lasater 2009<br>V Gajdos 2009<br>Erin Phinney Johnson 2009<br>Minna Huotilainen 2009<br>Chantal E H Dirix 2009<br>José F Fontanari 2009<br>Natalia Arias-Trejo 2009<br>Jonathan M Mansbach 2009<br>Danielle L Linklater 2009<br>Piers Dawes 2009<br>Maria Pino 2009<br>Koa Hosoki 2009<br>David J Lewkowicz 2009<br>Stefan Rücker 2009<br>M Proesmans 2009<br>Olivier Boucher 2009<br>Stanka A Fitneva 2009<br>James A Armontrout 2009<br>Sergio Morra 2009<br>Jessie Ricketts 2009<br>Tuomas Jartti 2009<br>Maurizio Bertollo 2010<br>G Montanari 2010<br>Cheol-Hong Min 2010<br>Patty Kostkova 2010<br>Chiara Cantiani 2010<br>Ana Paula Perez 2010							

Dheeraj Shah 2010<br>Yu Gao 2010<br>Michelle A Fortier 2010<br>Gaye Soley 2010<br>Eufemia Jacob 2010<br>Ayse Berna Anil 2010<br>M Moustaki 2010<br>A Schultz 2010<br>Alycia Cummings 2010<br>Yoram Greenstein 2010<br>C F B Murphy 2010<br>Robert Eisenberger 2010<br>Johannes C Ziegler 2010<br>Attilio L Boner 2010<br>Charlotte Giwercman 2010<br>Joshua Kuang-Chao Chen 2010<br>Shayne B Piasta 2010<br>Myoung Soo Kwon 2010<br>Nicole C Groskreutz 2010<br>Tracie B Mann 2010<br>Maria Isabel Ramos do Amaral 2010<br>Ross Flom 2010<br>Joseph P McCleery 2010<br>Bo L K Chawes 2010<br>Mariko Moher 2010<br>Marilene Danieli Simões Dutra 2010<br>Heather K J van der Lely 2010<br>A Bueso 2010<br>Christopher J Lemons 2010<br>M L García-García 2010<br>Anne-Claire Rattat 2010<br>Christy Shuk-kuen Chau 2010<br>Karen M Steele 2010<br>Regula Blaser 2010<br>David W Gerry 2010<br>Maciej Pronicki 2010<br>Luiza Bardi-Peti 2010<br>Cláudia da Silva 2010<br>Rachel Wu 2010<br>Luis Garcia-Marcos 2010<br>Weiguo Yao 2010<br>Monique Mommers 2010<br>Aaron Dennis 2010<br>Wieslaw Jedrychowski 2010<br>Samatha Sonnappa 2010<br>Jonathan C Kopelovich2010<br>Elena Plante 2010<br>Yun-Huei Ju 2010<br>Fengyu Cong 2010<br>Luis Garcia-Marcos 2010<br>Stavros Petrou 2010<br>Cristina Calvo 2010<br>Renzo Mora 2011<br>Anne Castles 2011<br>Edward M Hallowell 2010<br>Courtenay Frazier Norbury 2010<br>Kerstin H Kipp 2010<br>Michael Brambring 2010<br>Doris Luft Baker 2010<br>Juan E Jiménez 2010<br>T Jartti 2010<br>Paola Angelelli 2010<br>Silvia Bach 2010<br>Christopher W Robinson 2010<br>Tetsuro Nagasawa 2010<br>Hans Bisgaard 2010<br>Adriana Marques de Oliveira 2010<br>Turid Helland 2011<br>Michael T Willoughby 2011<br>YiWen Wang 2011<br>Ersoy Civelek 2011<br>Coralie Chevallier 2011<br>T Jartti 2011<br>Masafumi Sanefuji 2011<br>Marieke M van der Zalm 2011<br>Karin Harman James 2011<br>N M Iakovlev 2011<br>Xiaodong Lin 2011<br>Dongchuan Yu 2011<br>Holly L Storkel 2011<br>Fofi Constantinidou 2011<br>Cristina F B Murphy 2011<br>J Gavin Bremner 2011<br>Vijay A Mittal 2011<br>Fiona E Kyle 2011<br>E Ozkaya 2011<br>Julia Karbach 2011<br>Franziska Degé 2011<br>Martin Pusic 2011<br>Chiara Valeria Marinelli 2011<br>Laura Sangster 2011<br>Psyche Loui 2011<br>Carol Flexer 2011<br>Atsushi Isozaki 2011<br>Nina L Saine 2011<br>Cláudia Cardoso-Martins 2011<br>Hua-Chen Wang 2011<br>Tania Principi 2011<br>Plamenka Nanova 2011<br>Aini Ismafairus Abd Hamid 2011<br>Cheri C Y Chan 2011<br>Elham Ahmed Shaheen 2011<br>Ela Erdem 2011<br>T Liu 2011<br>Alberto Papi 2011<br>Marc Lievens 2011<br>Decio Medeiros 2011<br>Zhenkun Gou 2011<br>Jai Kumar Mahajan 2011<br>Khaled Jadid 2011<br>Cristina Gonçalves Alvim 2011<br>Constance Baldwin 2011<br>J Forster 2011<br>Panagiotis G Simos 2011<br>Mindy Sittner Bridges 2011<br>Jason D Yeatman 2011<br>Rachel Schmale 2011<br>Emily Mather 2011<br>Ana Esplugues 2011<br>Julie Sutton 2011<br>Per J Palmgren 2011<br>Laura L Grow 2011<br>Júlia Escalda 2011<br>Henrik Overödder 2012<br>Pierre-Alex Crisinel 2012<br>C H Razi 2012<br>H Peyton Young 2011<br>Johanna Goepel 2011<br>Morris Gordon 2011<br>Margaret M Swingler 2011<br>Emma Göksör 2011<br>Jeff T Larsen 2011<br>Monika Kast 2011<br>Robert S Zeiger 2011<br>Nadja Cristiane Lappann Botti 2011<br>Anton Aluja 2011<br>Tilbe Göksun 2011<br>Paul L P Brand 2011<br>Lisa Feigenson 2011<br>F Midulla 2012<br>D Mendis 2012<br>Lorri Centineo 2012<br>Hanako Yoshida 2012<br>S Orlandi 2012<br>Marc J Lanovaz 2012<br>Charlotte L Carp 2012<br>Markus Paulus2012<br>Hua-Chen Wang 2012<br>Judith A Gierut 2012<br>Y Uziel 2012<br>Ângela Pires 2012<br>Vesa Putkinen 2012<br>Norbert Maïonchi-Pino 2012<br>Fofi Constantinidou 2012<br>J Pellegrini-Belinchón 2012<br>Yuko Amimoto 2012<br>Vandana A Aspen 2012<br>Liesbeth Duijts 2012<br>Tracianne B Neilsen 2012<br>Brian E Anderson 2012<br>Allison M Fox 2012<br>Johannes C Ziegler 2012<br>Maureen W Lovett 2012<br>Rebecca F Walker 2012<br>Laura H F Barde 2012<br>Takanari Ikeyama 2012<br>R Kumar 2012<br>Stephen Montefort 2012<br>Kineret Sheinvald-Shusterman 2012<br>Andrew Hill 2012<br>Yueh-Hsiu Mathilda Chiu 2012<br>Katie Snyder 2012<br>Stephanie Burnett Heyes 2012<br>Jocelyne Just 2012<br>Paul Eggert 2012<br>Xin Lou2012<br>Dorit Ater 2012							

Markéta Caravolas 2012							
Charles Hulme 2012							
Samantha Johnson 2012							
Lucie Kappelle 2012							
Peter B Sullivan 2012							
George K Georgiou 2012							
J Gavin Bremner 2012							
Jason Debley 2012							
Ozge Yilmaz 2012							
S Ayari 2012							
Anne Martin 2012							
Kate E Norwalk 2012							
Tina M Grieco-Calub 2012							
Magdy Zedan 2012							
R Alexandre 2012							
Nanna Skytt 2012							
Eva M Syngelaki 2013							
Celeste Meijs 2013							
E R Gasenzer 2012							
Ross Flom 2012							
Noboru Takahashi 2012							
Cem Hasan Razi 2012							
Ana Dela Bianca 2012							
Sam Nordfeldt 2012							
Jason Young 2012							
E Kreiner-Møller 2012							
Mary Colleen Simonelli 2012							
Natasha Z Kirkham 2012							
Yufang Cheng 2012							
G Paul Amminger 2012							
Patrick Bonin 2012							
Przemko Kwinta 2013							
James R Engle 2013							
Renata Almeida Araujo Silvestre 2013							
Joshua A Lawson 2013							
Nelís Soto-Ramírez 2013							
Mara Trenta 2013							
Michael Potegal 2013							
Valerie Gay 2013							
S Caballero 2013							
Hongyan Sun 2013							
Eduard Schreibmann 2013							
Pooja S Tandon 2013							
Wim Van der Elst 2013							
Annemarie Kerkhoff 2013							
George N Konstantinou 2013							
Sophie La Vincente							
Ozge Yilmaz 2012							
Jose L Marroquin 2013							
William Hal Martin 2013							
Meredith Pike 2013							
Minna Lukkarinen 2013							
S Orlandi 2012							
Umit Murat Sahiner 2013							
Mikimasa Omori 2013							
Sabine Heim 2013							
Alfredo Cano Garcinuño 2013							
B J Kim 2013							
Hyekyun Rhee 2014							
S Caballero 2013							
Nicki Young 2014							
Marco A Valadares 2013							
Nora M Raschle 2014							
David J Lewkowicz 2013							
Anne C van der Gugten 2013							
Hongyan Sun 2013							
David Wertheim 2013							
Paul Silverston 2014							
Barbara Wagensveld 2013							
Virgil Zeigler-Hill 2013							
Adam N Sanborn 2013							
Jana Muenssinger 2013							
Weerasak Chonchaiya 2013							
Desiderio Passali 2013							
Mara Trenta 2013							
Isabelle Peretz 2013							
Fia ten Brink 2013							
Charlotte Giwercman Carson 2013							
Leher Singh 2014							
Mònica Guxens 2014							
Daniel M Connolly 2013							
Blake E Butler 2013							
Robert Smith 2013							
Michael Potegal 2013							
Markéta Caravolas 2013							
Valerie Gay 2013							
Juan Emilio Balinotti 2013							
Arno Steinacher 2013							
Beau Robertson 2014							
Katherine D Hoerster 2013							
Daniel C Hyde 2013							
Nadege Doignon-Camus 2013							
Kristen Swan Tummeltshammer 2013							
Steven S Robertson 2014							
Seunghyun Baek 2014							
Keiko K Fujisawa 2013							
Nelís Soto-Ramírez 2013							
Desmond W Cox 2013							
Cristina O'Callaghan-Gordo 2013							
Claudio Schvartsman 2013							
Lucie Bouvet 2016							
Erika A Henry 2014							
Alisha K Wackerle-Hollman 2015							
S Hélène Deacon 2014							
Yueh-Hsiu Mathilda Chiu 2014							
Joshua A Lawson 2013							
Angelina Paolozza 2014							
Erdem Topal 2013							
Giovana Serrão Fensterseifer 2013							
Wokie Weah 2013							
Jelka Tzschoppe 2014							
Gitte Falcher Madsen 2014							
Maiara Lanna Bouzas 2014							
Sébastien Pacton 2013							
Francesca Patria 2013							
Vesa Putkinen 2014							
Lorraine L T Cheung 2014							
Irit Bar-Kochva 2014							
John W O'Kane 2014							
Pastora Martínez-Castilla 2015							
Jean-Paul Collet 2014							
Khalifa Abdel Rahim Adam 2014							
Michael Mace 2014							
Prudence Allen 2014							
Claudia List Hilton 2014							
Olivia A A Costa Bessa 2014							
Jill Lany 2014							
Wen-jiao Zhou 2013							
Anja Schmitz 2014							
Johannes C Ziegler 2013							
Cynthia S Puranik 2013							
Eamonn Arble 2014							
Jeremy W Murphy 2014							
Ricardo Lopes 2014							
Jose A Castro-Rodriguez 2014							
Amee D Baird 2014							
Alfredo Cano-Garcinuño 2014							
Aya Takeyama 2014							
Antonio Clavenna 2014							
Gretchen Lefever Watson 2014							
Helen L Ferrett 2014							
Paul L P Brand 2014							
Laura Mihaela Trandafir 2013							
Meredith Peterson 2014							
Nathan H Clemens 2014							
Asa Neuman 2014							
Szilvia Biro 2014							
Kamini Ramdas Ilamkar 2014							
Nora Erkkola-Anttinen 2014							
Wendy J Ungar 2014							
Priya Jagota 2014							
Marjorie Beeghly 2014							
Dominique T Vuvan 2014							
André A B Varella 2014							
Soo Ji Kim 2015							
Fabio Midulla 2014							
Douglas B Petersen 2016							
Valentina Fantasia 2014							
Vishwanath Gowraiah 2014							
Karen M Steele 2014							
Li-Qin Chen 2015							
Pat Rojmahamongkol 2014							
Laura L Grow 2014							
A Tagarro 2014							
Laia Font-Ribera 2014							
Erin M Ingvalson 2014							
Eleanor B Peterson 2014							
Hanna Mulder 2014							
Susanna Esposito 2014							
Daniele Caligiore 2014							
Michael R DeBaun 2014							
Françoise Rochette 2014							
Arvind Sehgal 2014							
Patricia Aparecida Zuanetti 2014							
Mieke Vandewaetere 2015							
Hai Lee Chung 2015							
Nadja Althaus 2014							

Kiat Hui Khng 2014<br>Celeste Kidd 2014<br>Yuhe Zhang 2014<br>Metin Uysalol 2014<br>Joseph Vedora 2015<br>Tiffany Kodak 2015<br>Christian D Helfrich 2014<br>Clara E James 2015<br>Jamaree Teeratakulpisarn 2014<br>Patricia Roca 2014<br>Marta Krawiec 2015<br>Leslie R Bernstein 2014<br>Joanna Jacobus 2014<br>Katherine Rand 2014<br>Cynthia S Puranik 2014<br>Tyler L Renshaw 2015<br>Shirui Pan 2015<br>Chung-Il Wi 2015<br>Tuomas Jartti 2015<br>Annemarie Seither-Preisler 2014<br>Suzannah K Helps 2014<br>Christopher W N Saville 2015<br>Keila Alessandra Baraldi Knobel 2014<br>Katherine C Pears 2014<br>Xiaoli Shang 2014<br>Danielle C M Belgrave 2014<br>Susanna Esposito 2014<br>Caitlin H Delfs 2014<br>Noella A Piquette 2014<br>Karen A Gordon 2014<br>Lillian Sanchez Lacerda Moraes 2014<br>Sara Brolin Låftman 2015<br>G Brian Thompson 2015<br>P B Luz 2015<br>Isabel Cristina C Ferreira 2014<br>Paola Angelelli 2014<br>Anne van Bysterveldt 2014<br>Riitta Turunen 2014<br>Nichanan Ruangwattanapaisarn 2015<br>Antonios Marmarinos 2015<br>Lara J Pierce 2014<br>N N Zavadenko 2014<br>Tanatorn Tanantong 2015<br>Xiaoran Ma 2016<br>Keith J Zullig 2015<br>Veena Rajkumar 2014<br>Linda Lacina 2015<br>Irune Fernández-Prieto 2015<br>Mohamed Elgendi 2014<br>P Lajoie 2015<br>Lisa Cantrell 2015<br>Gitte Falcher Madsen 2015<br>Sharon K Hunter 2015<br>Priscila Cruvinel Villa 2014<br>Richard Ribón Fletcher 2014<br>Sophie Galer 2015<br>Ritva Torppa 2014<br>Julie E Dockrell 2015<br>H Irem Turkmen 2015<br>Andréa Huau 2015<br>Mirjana Kostić 2015<br>Lauren B Adamson 2015<br>Sarah C Creel 2015<br>Ernesta Paukste 2015<br>Joshua B B Garfield 2015<br>A Bercedo-Sanz 2015<br>Doris Nilsson 2015<br>A Borangiu 2014<br>Mallory J Quinn 2015<br>Paul J Turner 2015<br>Heather Kennedy 2015<br>Maimu Alissa Rehbein 2015<br>Michael Vassilyadi 2015<br>Ana Margarida Pereira 2015<br>Christian J Carlsson 2015<br>Annette M E Henderson 2015<br>Xiaoli Shang 2017<br>Rachel Schiff 2017<br>Jenni Heikkilä 2016<br>Jacki M Daniels 2016<br>Cristian Abelairas-Gómez 2015<br>B Giordani 2015<br>Kamaram Munira 2015<br>Mahdi Moeini 2015<br>M Morales 2016<br>Ping Zou 2016<br>Robert P Olympia 2016<br>Christopher J Lemons 2015<br>Yiguang Liu 2015<br>Adam T Tierney 2015<br>Kyle Hasenstab 2015<br>Nora Erkkola-Anttinen 2015<br>Bhamar Lal 2015<br>Claire Delle Luche 2015<br>Josefine Horbach 2015<br>Katharine Graf Estes 2015<br>Raffaella Nenna 2015<br>Minna Lukkarinen 2015<br>Danielle L Dupuis 2015<br>Isabelle Dautriche 2015<br>Wan-Yu Hung 2015<br>Colleen Cheek 2015<br>Javier Mallol 2016<br>Anna Ingeborg Petursdottir 2016<br>Kazuko Sugai 2016<br>K E van Wonderen 2016<br>Yiannis A Savva 2016<br>P Niemi 2015<br>Dean D'Souza 2015<br>Marta Navratil 2015<br>Chung-Il Wi 2015<br>Thalía Fernández 2016<br>Kacie C A Blackman 2015<br>C Kiese-Himmel 2016<br>Angeline Sin Mei Tsui 2016<br>Veit M Stoecklein 2015<br>B Nolan 2016<br>Jakob Stokholm 2016<br>Yue Yu 2016<br>Katie A McLaughlin 2016<br>Thomas Cook 2015<br>Ena Vukatana 2016<br>D A Young 2016<br>Petra Augurzky 2016<br>Robert D Friedberg 2016<br>Stacey L Burgess<br>Lara J Pierce 2015<br>Fatimah S Dawood 2016<br>Somaia Tawfik 2015<br>D A Ivanova 2015<br>Mirela Boscariol 2015<br>Christine Barton 2015<br>Tamer Abou-Elsaad 2016<br>Young Jin Ryu 2016<br>Richard J May 2016<br>Fleur Lejeune 2016<br>Marilyn Urrutia-Pereira 2016<br>Luciana Indinnimeo 2016<br>Kalina J Michalska 2016<br>Neha Thakur 2016<br>Sadako Vargas 2016<br>Huiquan Sun 2016<br>Kyle Hasenstab 2016<br>Plamen Bokov 2016<br>Katri Saarikivi 2016<br>Matthias Görges 2016<br>Beth L Green 2015<br>Erin E Flynn-Evans 2016<br>Giulia Cartocci 2015<br>Wolfgang Mastnak 2016<br>Maarten O Blanken 2016<br>Selda Hançerli-Törün 2015<br>Burak Erem 2016<br>Gregoire Benoist 2016<br>Derek Fish 2017<br>Daniel Dunea 2016<br>Ida M Bosga-Stork 2017<br>Juliana R Oliveira 2016<br>Wei-Ping Lan 2016<br>Wei-Ping Lan 2016<br>J Mishra 2016<br>Ansar U Ahmmed 2016<br>Sanne J H van Rooij 2017<br>Tian Gao 2017<br>Karen A Gordon 2016<br>Riitta Turunen 2016<br>Mohinder Sarna 2016<br>Alexandra L Quittner 2016<br>Joshua L Williams 2016<br>Perrine Brusini 2017<br>Katie M Pfister 2016<br>Shelley S Arnold 2016<br>Elina Naydenova 2016<br>M Willeboordse 2016<br>K Stenberg-Hammar 2016<br>L A Petitto 2016<br>Julia Hartkopf 2016							

John Woodward 2017<br>Tara Vongpaisal 2016<br>Maja Popovic 2016<br>Mohsin Ali Cheema 2016<br>Melissa K Ainsworth 2016<br>Anders Hurtig 2016<br>Marianne A Stephan 2016<br>Nina Hedayati 2016<br>Andrew Martin 2016<br>V Lezana 20117<br>Amy A Overman 2017<br>Henry Otgaar 2016<br>Alyssa Power 2017<br>Renuka Roche 2016<br>Sima K Ramratnam 2017<br>Sander Van de Cruys 2017<br>Firas Rinawi 2017<br>Annette Lohbeck 2017<br>Sharon Cameron 2015<br>Laura Toivonen 2016<br>Sukryool Kang 2017<br>Nelly D Saenen 2016<br>Gabriela R Gomes 2016<br>Daniel Dunea 2016<br>Qingguo Chen 2017<br>Kirsty M Ross 2016<br>K K Jasińska 2017<br>Ulrich Schroeders 2016<br>Jeanne L Shinskey 2017<br>A Teijeiro 2017<br>Jeremy Kawahara 2017<br>Ambra Nicolai 2017<br>Jeffrey S Larson 2017<br>Alyssa J Kersey 2017<br>Richard Baumgartner 2017<br>Jeremy M Law 2017<br>D C Moreira 2016<br>Elif Isbell 2016<br>Bo Chen 2016<br>Cristina Calvo 2017<br>Jessie B Northrup 2017<br>Marilyn G Boltz 2017<br>Nathalie Doorenweerd 2017<br>Meir Mei-Zahav 2017<br>Nicolas Wallaert 2017<br>Celine Delestrain 2017<br>Minghao Guo 2017<br>N Preston 2018<br>Nathan H Clemens 2018<br>Kyle Hasenstab 2017<br>Shilpa Dang 2017<br>Joshua A Lawson 2017<br>Ana Carla Leite Romero 2017<br>Riitta Turunen 2017<br>Anna Krzyżak 2018<br>Marius Baguma 2017<br>Minna Lukkarinen 2017<br>Jeanne Charles 2017<br>Anat Zaidman-Zait 2017<br>Pether Jildenstål 2016<br>Annamari Koistinen 2017<br>Prithi Sureka Mummidi 2017<br>A J Macnab 2018<br>Yu-Lin Cheng 2017<br>Valerie San Juan 2017<br>Tina L Samuels 2017<br>Maria Carla Spinosi 2017<br>Catherine E Laing 2017<br>Vassilios Argyropoulos 2017<br>Christina Y Tzeng 2017<br>George Georgiou 2017<br>John W McCarthy 2017<br>Arash Gharehbaghi 2017<br>Gmaan Alzhrani 2017<br>Janne von Koss Torkildsen 2018<br>Yuanhang Liu 2017<br>Zane Chou 2017<br>Flore Amat 2017<br>Karaked Chantawarangul 2018<br>Kristina Denisova 2017<br>Marco Flavio Michele Vismara 2017<br>Yerim Choi 2017<br>Ashley Anderson 2018<br>Maja Jurca 2017<br>Piush J Mandhane 2017<br>Martín Bedolla-Barajas 2017<br>Sven Braeutigam 2018<br>Annie Möller 2017<br>Rebecca Sims 2017<br>Julia Neily 2018<br>Ben D Spycher 2017<br>Ozlem Keskin 2018<br>Konstantin Sapozhnikov 2017<br>Jocelyn Gomez 2017<br>Ji Eun Soh 2017<br>M Dogru 2017<br>Mauricio Rocha Calomeni 2017<br>Laura Petrarca 2018<br>William Kovacs 2017<br>Olga Milczarek 2018<br>Letícia Gregory 2018<br>Tristan W Harding 2017<br>Tristan W Harding 2017<br>Eric Berg 2018<br>Önder Doksöz 2017<br>Ofer Golan 2018<br>Elika Bergelson 2017<br>B Christian 2017<br>Kenichi Tetsuhara 2017<br>Vijayalakshmi Easwar 2017<br>Hancan Zhu 2018<br>Jaswandi Tushar Pitale 2018<br>Gabriela Aída Szulman 2017<br>Kristen LaMarca 2018<br>Fabrizio Minichilli 2018<br>S J Foster 2018<br>Stephan D Ewert 2020<br>Xia Guo 2018<br>Peter Walker 2018<br>Qin Zhong 2018<br>Susanne Kutzora 2018<br>Christopher Papic 2019<br>C-I Wi 2018<br>Angela N Maupin 2019<br>Laura T Colman 2018<br>Norman D Cook 2017<br>Sau Kuan Cheong 2018<br>Carolyn Goldberg Butler 2018<br>Isabelle Roskam 2019<br>Sébastien Pacton 2018<br>Pierce Edmiston 2018<br>Kelli Cristina do Prado Côrrea 2018<br>Mohammed Khalifa 2017<br>Nienke M Scheltema 2018<br>Francina J Clayton 2018<br>Robert Bush 2018<br>Tracy L Kettering 2018<br>Andrea M Coverstone 2018<br>Romesh G Abeysuriya 2018<br>Saghafi Ellie 2018<br>Andreas Nickisch 2018<br>Waheed Atilade Adegbiji 2018<br>Catarina Vales 2018<br>Sandra Cano 2018<br>Yang Zhao 2018<br>Mads Poulsen 2018<br>Smadar Z Patael 2018<br>Antonio Pizzulli 2018<br>Brittney M Donovan 2018<br>Tanja Linnavalli 2018<br>Anna S Gellert 2018<br>Matteo Bonato 2018<br>Hanna Keren 2018<br>Margriet van Iersel 2018<br>Takashi Furuta 2018<br>Kedir N Turi 2018<br>R J Lund 2018<br>Shelley S Arnold 2018<br>Nils Hampe 2019<br>Orianne Dumas 2019<br>Merel M VAN Goch 2019<br>Paul Walsh 2018<br>T Mikeladze 2018<br>Huiming Sun 2018<br>Jan-Henning Ehm 2019<br>Paul Ibbotson 2018<br>Fleur Lejeune 2019<br>Annamari Leino 2019<br>Marcelo Zanchetta do Nascimento 2018<br>Brian O'Hara 2018<br>Mary Rudner 2018<br>Bryan Weichelt 2018<br>Priyanka Patel 2018<br>Conor Fahy 2019<br>Lori M Curtindale 2019<br>Cheol-Hong Min 2018<br>K Kaminishi 2018							

Cameron Hoerig 2019<br>Christopher W Robinson 2019<br>Julie L Darbyshire 2018<br>Séverin Lemaignan 2018<br>Luca Cilibrasi 2018<br>Jaimie K Beveridge 2018<br>Taku Obara 2018<br>Caroline Dutil 2018<br>Travis J Saunders 2018<br>Mark S Tremblay 2018<br>Patricia E Longmuir 2018<br>Robert M T Madiona 2018<br>Pamela Zúñiga-Bello 2019<br>Maeve R Boylan 2019<br>Theresa W Guilbert 2019<br>Emire Mırıcı 2018<br>John W McCarthy 2019<br>Weihong Han 2019<br>Katherine S Davlantis 2019<br>Sandra R Gomes 2019<br>Carol Cox 2018<br>Burcu Sarı 2019<br>Angela M AuBuchon 2019<br>Samuel H Forbes 2019<br>Eleni Athanasiadou 2018<br>Diane M Gray 2019<br>Alessia Barlotta 2019<br>Tong Gou 2019<br>Sarah N Adams 2021<br>Sara Crawford 2019<br>Bárbara Antunes Rezende 2019<br>Jessica P Uy 2019<br>Robin Marlow 2019<br>Natalia V Oster 2019<br>Tomasz Grzywalski 2019<br>Christiane Patzwald 2019<br>Hamid Shokoohi 2019<br>Ceyda Oksel 2019<br>Rajay Rampersad 2019<br>Qing Lu 2019<br>M Arrais 2019<br>Elizabeth Randell 2019<br>Conrad Perry 2019<br>Lena van der Velde Kremin 2019<br>Tingting Zhao 2019<br>Maud J A Raaymakers 2019<br>Y J Jiang 2019<br>Lee Jollans 2019<br>Wayne W Fisher 2019<br>Armin Eftekhari 2020<br>Christopher W Roy 2019<br>Justin Chan 2019<br>Karin Yaacoby-Bianu 2019<br>Alaa Mohamed AbdelKader 2019<br>Silvia Bonacina 2019<br>Susanne Nemholt 2020<br>A Della Volpe 2019<br>Hongquan Qu 2019<br>Hongmin Gao 2019<br>Paul van Schaik 2019<br>Hanna Kim 2020<br>Elizabeth C Parsons 2019<br>Daphne A Henry 2019<br>Nicole M Hanney 2019<br>Kaixiang Yang 2020<br>Gaobo Liang 2020<br>Libby Azaryahu 2020<br>Maria E Powell 2019<br>Nina Politimou 2019<br>Muhammad Arsalan 2019<br>Xiao-He Yu 2019<br>Katie L Burkhouse 2019<br>Xin-Hui Yuan 2020<br>Tabea Brick 2019<br>Vesa Putkinen 2019<br>Lyndsay A Fairchild 2020<br>Kangkang Liu 2020<br>James Negen 2019<br>Juli Coffin 2019<br>Sabrina Turker 2019<br>Veronika Vilgis 2020<br>A Teijeiro 2020<br>Sanjay Kumar Roy Chowdhury 2019<br>Leah Cuthbertson 2019<br>Shintaro Katayama 2020<br>Teresa Y C Ching 2019<br>Irene Altarelli 2020<br>Joanna J Parga 2020<br>Changde Cheng 2019<br>Lana Dos Santos Martins 2021<br>Sumarga H Suanda 2019<br>Luka Gligic 2020<br>Giovanni Anobile 2019<br>Satoshi Kida 2020<br>Yuichi Kama 2020<br>Zonghai Zhu 2020<br>Maria Arroyo 2020<br>Rebecca Treiman 2020<br>Xiaoyan Zhang 2020<br>Michael Gaies 2019<br>Laure-Hélène Canette 2020<br>Julia Alfonso 2020<br>Xinmin Tao 2020<br>Désirée Larenas-Linnemann 2020<br>Ke Song 2019<br>Francisco J Ruiz-Martínez 2020<br>Y Wang 2020<br>Chisha T Sikazwe 2019<br>Rakshita Gokula 2019<br>Nurcicek Padem 2019<br>Hussein Rizal 2019<br>Yang Lu 2019<br>Stephen C Bunt 2021<br>Samuel David Jones 2020<br>Maya Harel-Sterling 2020<br>Eric N Minor 2020<br>Biyun Yang 2020<br>Maleewan Kitcharoensakkul 2020<br>Nienke van Atteveldt 2020<br>Xiaoxiao Li 2020<br>Bronwyn K Brew 2019<br>Matea Deliu 2020<br>Daisy R Singla 2020<br>Nuria Gutiérrez 2020<br>Zohreh Hamedani 2019<br>Nishit H Patel 2020<br>Zhao Qing 2019<br>Amr S Soliman 2021<br>Sharon Cameron 2020<br>Thomas G Muir 2020<br>Patrick Quercia 2020<br>H Broadbent 2020<br>Carlos Milovic 2020<br>Felix G Knorr 2020<br>Daniela M Ribeiro 2020<br>Gabrielle Appleford 2019<br>Ivar Snorrason 2019<br>Chén C Kenyon 2020<br>Alvin M Matsumoto 2020<br>Giulio Gabrieli 2020<br>Alexander Prehn-Kristensen 2020<br>Saeid Parvandeh 2020<br>Sasawan Chinratanapisit 2023<br>Diana Malaeb 2020<br>Katharine Leiter 2020<br>Cathryn J Luria 2020<br>Ilson Meireles-Neto 2020<br>Martina Caccia 2021<br>Stéphanie Lejeune 2020<br>Valbona Gashi 2020<br>Zhongliang Wang 2020<br>Avantika Mathur 2020<br>Ville-Pekka Seppä 2020<br>Peter Kochunov 2022<br>Rajesh Sanjeevi 2020<br>Magd Ahmed Kotb 2020<br>Christopher Pennell 2020<br>Colin M E Halverson 2020<br>Ta-Wei Tang 2020<br>Richard Lakeman 2020<br>Betel Yibrehu 2020<br>Junshen Xu 2019<br>David F Van Komen 2020<br>Rebecca O'Donovan 2020<br>Cristina Calvo 2020<br>Ramtin Zargari Marandi 2020<br>Juan Luis Fernández-Martínez 2020<br>Muhammad Fazal Ijaz 2020<br>Ruiqing Zheng 2020<br>Daniel Dunea 2020<br>Yuanyuan Wang 2020<br>Nicolas Gille 2020<br>Jihye Rhee 2020<br>Sharon McMurray 2020<br>Sharon McMurray 2020<br>Amy A Schultz 2020<br>Huan Minh Luu 2021							

Aleksandar Sovtic 2020							
Alexander E White 2020							
Yvonne Bingham 2020							
Emmanuel Ahishakiye 2020							
Ana Sucena 2020							
Yuzhu Ji 2020							
Margaux Lê 2021							
Arash Gharehbaghi 2020							
Hoi See Tsao 2021							
A Brooks Bowden 2020							
Deisiane Amorim da Silva 2020							
Molly Farry-Thorn 2020							
Brian A Sharpless 2020							
Tobias Rydén 2021							
Katie Alcock 2020							
Chizu Habukawa 2020							
Hakan Guvenir 2021							
Arianna Di Stadio 2020							
Florian Kromp 2020							
JoAnne E Natale 2021							
Heidi Makrinioti 2020							
Shelley Xiuli Tong 2020							
Alayna P Tackett 2020							
Mateusz C Florkow 2020							
Monika M Derrien 2020							
Jason D Kelly 2020							
Wayne Richards 2020							
Raquel Rodrigues Dos Santos 2020							
Katharine Graf Estes 2021							
Ghazal Aghagoli 2021							
Madeleine Hui 2021							
Adele Diamond 2020							
Rebecca Singer 2020							
Khaled A Abdel Baseer 2021							
Elisabet Engström 2020							
Katerina Pavlos 2021							
Fawen Zhang 2021							
Samantha Bergmann 2021							
Xin Cui 2020							
Shahed Ahmed 2021							
Yao Sui 2020							
Anita Harrewijn 2021							
Xiaoyan Zhang 2021							
Mridula Sharma 2020							
Pascale M J Engel de Abreu 2020							
Jie Zhao 2020							
Yayi Zhao 2021							
Jennifer A Silvers 2021							
Howard H F Tang 2021							
Florian B Pokorny 2020							
Yanyun Tao 2020							
Xiaoxiao Li 2020							
Luca Oppici 2020							
George Athanasopoulos 2021							
Kedir N Turi 2021							
Jingyang Li 2021							
Ilvana Dzafic 2021							
Jessica M Kramer 2021							
Toshimune Kambara 2021							
Mark A Connelly 2021							
Olga Lazareva 2021							
Nils Rüllmann 2020							
Timm Rosburg 2021							
Chuanji Gao 2021							
Juliano De Souza Gaspar 2020							
Hong Zeng 2020							
Si Young Yie 2020							
John Eichwald 2020							
Elisabet Engström 2021							
Emily Su 2020							
Lalith Kumar Shiyam Sundar 2021							
Jukka Ranta 2021							
Nathan R Huber 2021							
Hasitha Wimalarathna 2021							
T B Neilsen 2021							
Samuel R Mathias 2021							
Rosa Rodríguez-Fernández 2021							
Lara J Pierce 2021							
Joram van Driel 2021							
Hongxin Wang 2021							
Dominik Hangartner 2021							
Linda K Sundermann 2021							
Hiroki Higuchi 2021							
Nandini Arul 2021							
N Jeremy Hill 2021							
Nicki Hartney 2021							
Alexandre Pitti 2021							
Ting Su 2021							
Lihua Yang 2021							
Chiara Valeria Marinelli 2021							
Catharina Janner 2021							
Ryan A Stevenson 2021							
Alessandra Schneider 2021							
Wellington Fernando da Silva Ferreira 2021							
Sergi Gómez-Quintana 2021							
Stéphanie Bourion-Bédès 2021							
Alok Kumar 2021							
Qiaozhen Zhang 2021							
Alex Gileles-Hillel 2021							
David J Forman 2021							
E K Schworer 2022							
Mary Halbur 2021							
Shao-Lun Lu 2021							
Felipe Beijamini 2021							
Sarah Suárez 2021							
Sainan Chen 2021							
Yunquan Zhang 2021							
Rong Guo 2021							
Giulia Lazzaro 2021							
Laurent Demany 2021							
David F Van Komen 2021							
Sweta Mohanty 2021							
Mahdieh Dashtbani Moghari 2021							
Christina Sarosiek 2021							
Ahnjili ZhuParris 2021							
Srivathsa Pasumarthi 2021							
A AbdelMassih 2021							
Ziqiang Lin 2021							
Pirko Tõugu 2021							
Chizu Habukawa 2021							
Alysha D Gilmore 2021							
Nicola Ullmann 2021							
Bochun Wang 2021							
Steen Rasmussen 2021							
Yue Gu 2021							
Antonio García-Domínguez 2020							
Annette Sundqvist 2021							
Yeong-Hyeon Byeon 2021							
Laura K Young 2021							
Vladimir Gligorijević 2021							
Eun Cho 2021							
Buendía Jefferson Antonio 2021							
Gorka Fraga-González 2021							
Sarah Al-Salim 2021							
Katherine Finn Davis 2021							
Rainer Boegle 2021							
Shen-Hao Lai 2021							
Polly F M Robinson 2021							
Teresa de la Calle Cabrera 2021							
Milovan Regodić 2021							
Sebastian Tschauner 2021							
Vesa Putkinen 2021							
Gabrielle R Merchant 2021							
Camilla L Fitjar 2021							
Jingyang Li 2021							
Adam Tierney 2021							
Jordi Julvez 2021							
Pekka Hurme 2021							
Sümeyye Koşkulu 2021							
Mehmet Kılıç 2021							
Emma K Ward 2022							
Jihang Sun 2021							
Michael Zeiler 2021							
Jihang Sun 2021							
Stephanie J W Shoop-Worrall 2021							
Fernando M Calatayud-Sáez 2021							
Silvana Mantovani 2021							
Silvia Bonacina 2021							
Jingjing Tang 2021							
Davood Karimi 2021							
Camilo Gouet 2021							
Laura Del Hoyo Soriano 2021							
Shruti Balvalli Deshpande 2021							
Jihang Sun 2021							
Allison Frost 2021							
Max H Siegel 2021							
Tineke Grent-'t-Jong 2021							
B T Balamurali 2021							
Maria Chifa 2021							
Kelsey Eitel 2022							
Aivett Bilbao 2022							
Xuan Li 2021							
Wei-Lun Chung 2021							
Emad A Mohammed 2021							
Annina Fahr 2021							
Lei Wu 2021							
Jean Ecalle 2021							

Nicholas Cothros 2021<br>Che-Chou Shen 2021<br>Madeline Hallewell 2021<br>Sara Momtaz 2021<br>Rebecca A Marks 2022<br>Avirath Sundaresan 2021<br>Sebastian Ludyga 2022<br>Jiachao Wu 2021<br>Michaël Sauthier 2021<br>Yasunori Nagayama 2021<br>Femke Vanden Bempt 2021<br>Pailin Yooma 2022<br>Jizuo Li 2021<br>Christian Rosas-Salazar 2022<br>Gyu-Ho Shin 2021<br>Nishit H Patel 2021<br>Aric F Logsdon 2022<br>Ting Wang 2021<br>Jihang Sun 2021<br>K L Purves 2023<br>Christian D Escobar-Amado 2021<br>Christian D Escobar-Amado 2021<br>Johannes Leuschner 2021<br>Davood Karimi 2021<br>Hyeon Ki Jeong 2022<br>Ebrahim Mohammed Senan 2021<br>R M Price 2021<br>Yao Sui 2021<br>Daniel C Whittingslow 2020<br>Nicole Wetzel 2021<br>Giulia Lazzaro 2021<br>Csaba Kertész 2021<br>Liugang Gao 2021<br>Dale Till 2021<br>Ágnes Lukács 2021<br>Zihao Sun 2021<br>Melkamu Hunegnaw Asmare 2021<br>Sandra L Turner 2022<br>Haesung Yoon 2021<br>William La Cava 2021<br>Hayder Mohammed Qasim 2021<br>Abdul Karim 2021<br>Lucas Vieira Lacerda Pires 2021<br>Q L Wang 2021<br>Jihang Sun 2022<br>Joseph D Olson 2022<br>Zhen Hadassah Cheng 2023<br>Sejal Saglani 2021<br>Marco A Formoso 2021<br>Nazareno Massara 2021<br>Carla J Greenbaum 2021<br>Daria F Kleeva 2022<br>Zai Ru Cheng 2022<br>Zai Ru Cheng 2022<br>Hongxia Luo 2022<br>Xuanyu Shan 2022<br>Fei Feng 2022<br>Matthijs D Kruizinga 2022<br>Stefanie Stadler Elmer 2021<br>Alexis Laurent 2021<br>Ting Su 2022<br>Jorge Oliveira 2022<br>Laura Petrarca 2022<br>Joshua R Harper 2021<br>Takaya Satoh 2022<br>Samantha Major 2022<br>Jia Liu 2022<br>Richard J Addante 2021<br>Christina Pirner 2022<br>Samantha F Harkus 2021<br>Elli Spyropoulou 2023<br>Yawen Yang 2021<br>J A Castro-Correa 2021<br>Xiaoan Yan 2021<br>Franca Rusconi 2022<br>Jordan Ezell 2022<br>Anastasia Filiou 2022<br>Zixun Huang 2022<br>Qian Guo 2022<br>Vanessa Foot-Seymour 2022<br>Kilian Knauth 2022<br>Laura Del Hoyo Soriano 2021<br>Christian Sunday Ugwuanyi 2022<br>Valentin Bégel 2022<br>Amir Besharat 2022<br>Narges Moradi 2022<br>Zicong Ge 2022<br>Jilei Lin 2021<br>Xuanyu Shan 2022<br>Xuanyu Shan 2022<br>Rebecca Treiman 2022<br>Chuang Wang 2021<br>Constantinos Kotsapas 2022<br>David Doan 2022<br>Nadja Cristina Furtado Back 2021<br>Lester Darryl Geneviève 2022<br>David S Mather 2022<br>Xin Duan 2022<br>Yuichi Kama 2022<br>Shikha Saxena 2022<br>Chih-Wei Lin 2022<br>Arash Gharehbaghi 2022<br>Hyoung Suk Park 2022<br>Xiangjun Wu 2022<br>Rodrigo San-Martin 2022<br>Xu Han 2021<br>Marjan Tariverdi 2022<br>Sean W Flannery 2022<br>Umarani Nagavelli 2022<br>Lucas A Barnes 2022<br>Caroline P Lubert 2022<br>Nimu Yuan 2022<br>Yu-Hao Liao 2022<br>Fan Rao 2022<br>Fayez Alharbi 2022<br>Bingshan Hu 2022<br>Yasunori Nagayama 2022<br>Irene van Kamp 2021<br>Maryam Ghorbani 2022<br>Zhichao Xia 2022<br>Salahuddin Ahmed 2022<br>Zhengen Wu 2022<br>Hailiang Sun 2022<br>O-Joun Lee 2022<br>Yichen Sun 2022<br>Huixian Zhang 2022<br>Danna Oomen 2022<br>Yongjun Zhang 2022<br>Eino Partanen 2022<br>Linn Damsgaard 2022<br>Yu Guan 2022<br>Luigi Matera 2022<br>Shujuan Mao 2022<br>Manoj Diwakar 2023<br>Ilona Kovács 2022<br>Hua-Chen Wang 2022<br>Bieke Tack 2022<br>Joel Janssen 2022<br>Camila Zugarramurdi 2022<br>György Hámori 2022<br>H H S Lam 2022<br>Stéphanie Bourion-Bédès 2022<br>Alperen Degirmenci 2022<br>Hai Cao 2022<br>Márcio L B Lopes Jr 2022<br>Alex J Warr 2022<br>Irene Tzovara 2022<br>Annette Sundqvist 2022<br>Claire Monroy 2022<br>James W Madole 2022<br>Martin Eklöf 2022<br>Fuquan Deng 2022<br>Meng Jiao 2022<br>T R Mahesh 202<br>Jianhong Zhang 2022<br>Elijah Flores 2022<br>Yijing Guo 2022<br>Zhiwei Huang 2022<br>T R Mahesh 2022<br>Hedan Chen 2022<br>Xin Dong 2022<br>Benjamin Ambrosio 2022<br>Keren Lehavot 2022<br>Yan Wang 2022<br>A Ankeeta 2022<br>Gail Gillon 2023<br>Kara L Kerr 2022<br>Milad Moradi 2022<br>Wookon Son 2022<br>Joseph C Y Lau 2022<br>Nils Rüllmann 2022<br>Yang Li 2022<br>Kun Zhang 2022<br>Silja Luotonen 2022<br>Kui Ding 2022<br>Rita Monteiro 2022<br>Rachel M Heacock 2022							

Syed Ali Yazdan 2022<br>Longzhao Huang 2022<br>Feng Duan 2022<br>Silpthai Thamruangrit 2022<br>Xuan Liu 2022<br>Benjamin W Frush 2022<br>Matthew J Urban 2022<br>Sibel Kayaaltı-Yüksek 2022<br>Yuliang Huang 2022<br>Tracey L Sletten 2022<br>Malte Sieren 2022<br>Adel Akbarimajd 2022<br>Hyeong-Ryeol Baek 2022<br>Ashkan Alvand 2022<br>Yuxuan Zhao 2022<br>Ramin Afshari 2022<br>Jarosław Żygierewicz 2022<br>Qiting Long 2022<br>Guangyi Chen 2022<br>Jane Saldanha 2022<br>Nicola Magnavita 2022<br>Tao Shou 2022<br>Daniel J Shapiro 2022<br>Anne Kotaniemi-Syrjänen 2022<br>David Wertheim 2022<br>Anna Crist Benson 2022<br>Connie Qun Guan 2022<br>Guiping Yu 2022<br>Gayatri Deshmukh 2022<br>Myrtha E Reyna 2022<br>Yuxiao Huang 2022<br>Gabriele Chierchia 2023<br>Shamsa Zafar 2022<br>Soo-Hyun Kim 2023<br>Katrina Pedersen 2021<br>Ozgu Aydogdu 2022<br>Mohammed J Abdulaal 2022<br>Jiangeng Chang 2022<br>Raymond J So 2022<br>Kanako Odagiri 2022<br>Qing Zhang 2022<br>N V L M Krishna Munagala 2022<br>Najibeh Atazadeh 2022<br>Jennifer M Becker 2022<br>Siqi Liao 2022<br>Andrew W Manigault 2022<br>Denise M Werchan 2022<br>Kun Hee Kim 2022<br>R Yates Coley 2022<br>Ravit Cohen-Mimran 2022<br>Cheng Zhao 2022<br>Yuri G Pavlov 2023<br>Miguel Valença 2022<br>M Filippa 2022<br>Peter C Raffalt 2023<br>Bo Wang 2022<br>Lu Li 2022<br>Chuansha Wu 2022<br>Anuradha Thakare 2022<br>Steffen A Herff 2023<br>Jiyoung Park 2022<br>Hermundur Sigmundsson 2022<br>Hao Xiong 2022<br>Alice Masini 2023<br>Haixu Yang 2023<br>Ruiwen Dong 2022<br>Amy T Edmonds 2022<br>Rakshita Gokula 2022<br>Benjamin Crysup 2023<br>Jiaxian Shen 2022<br>Tanja Linnavalli 2022<br>Pekka Hurme 2022<br>Minjuan Zhu 2023<br>Jolinda Smith 2022<br>Long Zhang 2022<br>Jahangir Esfandiari 2022<br>Roxane S Hoyer 2023<br>Yinsheng Chen 2022<br>Jacob Kullberg 2022<br>Yue Li 2022<br>Xiang Ren 2024<br>Natasha Janzen Ulbricht 2023<br>Andrew W Manigault 2023<br>Mingfu Nuo 2023<br>Debbie Zhao 2023<br>D Papp 2023<br>Yuanhao Huang 2023<br>Cui Fu 2022<br>Jingyu Gu 2023<br>Erin Conwell 2022<br>David C Morgenroth 2023<br>Emily Cary 2024<br>Jung Hyuk Lee 2022<br>Sirma Mileva 2022<br>Beom Joon Kim 2022<br>Junxiao Wang 2022<br>Jin Liang 2022<br>Syed Mohsin Ali Shah 2022<br>Risha Irvin 2023<br>Leila M Larson 2023<br>Sonu Acharya 2023<br>Zhili Wang 2023<br>Tiantian Wang 2023<br>Xiangji Guo 2023<br>Seyed-Ali Sadegh-Zadeh 2023<br>Yanmei Hu 2023<br>Haocheng Zhu 2023<br>Leonardo Martinez 2023<br>Brigitte Eisenwort 2023<br>Pil-Hyun Jeon 2023<br>Adrian Dybfest Eriksen 2023<br>Ho-Chang Kuo 2023<br>Yuan Li 2022<br>Sarah Thomas 2023<br>Ágnes Lukács 2023<br>Christine P Shen 2023<br>Yibing Zhu 2023<br>Natalie Rhodes 2023<br>Ellin Simon 2023<br>Kartik K Iyer 2023<br>Wei Zhang 2023<br>Zhenwei You 2023<br>Anna E Rueda 2023<br>Julie Nyholm Kyvsgaard 2023<br>Emma J Carpendale 2023<br>Plamen Bokov 2023<br>Jordan F Hastings 2023<br>Mahesh Thyluru Ramakrishna 2023<br>Pengpeng Jia 2023<br>Joshua Eisenstat 2023<br>Derek Long 2023<br>Nim Lee 2023<br>Zhihao Wang 2023<br>Di Yuan 2023<br>Sarah Kristine Nørgaard 2023<br>Aaron P Turner 2023<br>Ying Guo 2023<br>Edward Parkinson 2023<br>Prithvi Ravi Kantan 2023<br>Sarah Nazzari 2023<br>Stefan P Ewers 2023<br>Peng Gao 2023<br>Foram Joshi 2023<br>Tracy A Cameron 2023<br>Xiaoyang Zou 2023<br>Dongmin Huang 2023<br>Shota Ichikawa 203<br>Vick Lau 2023<br>Hongbo Yang 2023<br>Vahid Ganjalizadeh 2023<br>Khaled M Alalayah 2023<br>Nathan E Cook 2023<br>Yuhui Ma 2023<br>Elizabeth V Edgar 2023<br>Chang Sun 2023<br>Nai-Wen Chang 2023<br>Chengan Yuan 2023<br>Enrique A Navarro 2023<br>Maria Nicastri 2023<br>Xiaoyan Zhang 2023<br>Li Zhang 2023<br>Dong Hwan Kim 2023<br>Laure F Pittet 2023<br>Xiangjia Meng 2023<br>Elsayed E A Hassanein 2023<br>Mahdieh Dashtbani Moghari 2023<br>Joseph J Shenker 2023<br>Justin M Jeffers 2023<br>Cenyi Yang 2023<br>Sophia T Merrifield 2023<br>Felix Q Jin 2023<br>Jose Uberos-Fernández 2023<br>Tobias Weinmann 2024<br>David A Haslett 2023<br>Helga S Thórsdóttir 2023<br>Stephen R Dennison 2023<br>Philipp Thölke 2023							

Andrey Zhdanov 2023							
Heikki Lyytinen 2023							
Érica Prates Krás Borges 2023							
Nam Heon Kim 2023							
Keren Aridor 2023							
Deepak Jain 2022							
Zhili Wang 2023							
Louis A Gomez 2023							
Engin Melekoglu 2022							
Quentin Goossens 2023							
Senik Matinyan 2023							
Hengyu Man 2023							
Simone Dobbelaar 2023							
Peng Liu 2023							
Jo Fletcher 2023							
Chee Chin Lim 2023							
Tao Wang 2023							
Christine Bridges 2023							
Elda Fischi-Gomez 2022							
Youngwook Lyoo 2023							
Kehui Zhang 2023							
Maria P Dimopoulos 2023							
Gaurav Aggarwal 2023							
Inbal Raviv 2023							
Qiang Liu 2023							
Fazli Khaliq 2023							
Tianzhuo Liu 2023							
Amanda Fernandes 2023							
Youness Arjoune 2023							
Jiding Zhai 2022							
Stephanie W Y Chan 2023							
Adrian O'Dowd 2023							
Katrina Pedersen 2023							
Leann Fay Cyr 2024							
Yesim Saglam 2023							
K T Chen 2023							
Laura Beth Kalvas 2023							
Maria Teresa Gandia-Ferrero 2023							
Amanda Saksida 2024							
Maral Kasiri 2023							
M Priyadharshini 2023							
Nicolas Lynch-Aird 2023							
Raymond J So 2023							
Miroslava Migovich 2023							
Zongpai Zhang 2023							
Suchen Li 2023							
Matthew Rosenblatt 2023							
Julian Fox 2023							
Zhenzhong Gan 2023							
Carolyn A Fan 2023							
Peipei Li 2023							
Suraj Achar 2023							
Rachael A Batabyal 2023							
Chiara Visentin 2023							
Yu Luo 2023							
Shijia Li 2023							
Essam Abdellatef 2023							
Mari D Takashima 2023							
Peisheng Zeng 2023							
Renu Thakur 2023							
Ali Ebrahimi 2023							
Amitai Abramovitch 2024							
Yani Qiu 2023							
Kristine Anthony 2024							
Ana Sucena 2023							
Sunghoon Im 2023							
Zahra Hosseini Dastgerdi 2023							
Di Xiao 2023							
Liwei Deng 2023							
Kang Yi 2023							
Meiyi Zhu 2023							
Roneel V Sharan 2024							
Daria Kostanian 2023							
Mingxia Zhang 2024							
Ziqiang Ye 2024							
Tatsuya Daikoku 2023							
Ryo Kamizaki 2023							
Shannon Proksch 2023							
Julie A Beans 2023							
Serge Didenko Vasylechko 2024							
Sérgio Kakuta Kato 2023							
Takayuki Kitazawa 2024							
Ruben Domingues 2023							
Roneel V Sharan 2023							
Md Hassanuzzaman 2023							
Jeffrey A Barahona 2023							
Mattia Pugliatti 2023							
Jin-Han Lee 2023							
Yangqing Ye 2023							
Samantha Pegg 2024							
Katherine R Gordon 2024							
Nicholas Murphy 2024							
Lindsay A Jibb 2023							
Yu-Ting Tsai 2023							
Julie Nyholm Kyvsgaard 2024							
Lara Teheux 2024							
Shunsuke Yamada 2023							
Anne-Marie Chang 2024							
Weijie Jia 2024							
Priyanka Khante 2023							
Bryce E Hedelius 2024							
Qi Sun 2024							
Neha Rajappa 2023							
Abdulaziz Kebede Kassaw 2023							
Zeliha Kilic 2023							
Marica Muffoletto 2023							
Sujay Nagaraj 2023							
Sujay Nagaraj 2023b							
Isabelle Coenen 2024							
Seyed Vahid Alavi Nezhad Khalil Abad 2023							
Pierangela Presta 2024							
Giorgia Bussu 2023							
Annapurna Kala 2023							
M A Ganaie 2024							
Gaurav Kumar Nayak 2024							
Ziping Liang 2024							
Isabel J Hardee 2024							
Po Yun 2024							
Emma Roche 2024							
Jeff Joseph 2024							
Hauwa Ahmad Amshi 2024							
Inés Gonzalez Pepe 2024							
Yohan Jun 2024							
Luigi Grisoni 2024							
Xiaodong Song 2024							
Maryam Mehdizadeh 2024							
Aliaa Gouda 2024							
Yalin Tian 2024							
Andrea Sánchez-Corzo 2024							
Zehan Liu 2024							
Ping He 2024							
Xiangyun Zhao 2024							
Rui Wang 2024							
Kunpeng Li 2024							
Mélanie Lévesque 2024							
Naiqi G Xiao 2024							
Phil Birch 2024							
Yi Mu 2024							
Wenhao Zhang 2024							
Zakieh Hassanzadeh 2024							
Barsha Abhisheka 2024							
Haoyan Li 2024							
Alice Desclaux 2024							
Bengi Gül Türk 2024							
Miguel Navarrete 2024							
Adel Alharbi 2024							
Zhijiang Tang 2024							
Mitchell C Cutler 2024							
Danyal Khan 2024							
Fei Kong 2024							
Giulia Orioli 2024							
Nuo Xu 2024							
Hyunju Lee 2024							
Maja Sidstedt 2024							
Raúl Fernández-Ruiz 2024							
Aya Konno-Yamamoto 2024							
Binjie Hou 2024							
Lior Abramson 2024							
Premendra Kumar Singh 2024							
Huien Zhou 2024							
Jakub Kopal 2024							
Henrik Hellqvist 2024							
Abigail L Noyce 2024							
Ning Li 2024							
Natalia Andrienko 2024							
Rea Rantanen 2024							
Francisco Barbosa Escobar 2024							
Jean-Baptiste Guimbaud 2024							
W Gerald Teague 2024							
Christian Kapper 2024							
Hanna Bugler 2024							
Chao Gao 2024							
Alexandros Emvoliadis 2024							
George Zhou 2024							
Maria Angela Tosca 2024							
Liuliu Shi 2024							
Nicole Polara 2024							

Yang Ying 2024<br>Toni A May 2024<br>Xingqi Wang 2024<br>Hongmei Hu 2024<br>Zoe J Ryan 2024<br>Laura Santos 2024<br>Qiong Liu 2024<br>Zhenglin Pan 2024<br>Zhanhui Hu 2024<br>Maryam Mehdizadeh 2024<br>Yuheng Liao 2024<br>Luca Santini 2024<br>Jianhua Zhao 2024<br>David Lo 2024<br>Guanglei Yu 2024<br>Rodrigo M Carrillo-Larco 2024<br>Pekka Hurme 2024<br>Yang Yi Poh 2024<br>Ida A P A Crisdayanti 2024<br>Xiaohong Wu 2024<br>Deniz Tuz 2024<br>Tahsin Mullick 2024<br>Taofeng Xie 2024<br>Adrianne K Nelson 2024<br>Heidi Makrinioti 2024<br>Youquan Deng 2024<br>Sandra Fernandes 2024<br>Tian Tian 2024<br>Philip Capin 2024<br>Sally Richmond 2024<br>Hiroyuki Mochizuki 2024<br>Lili Cai 2024<br>Nataşa Ganea 2024<br>Wensi Wu 2024<br>Zuhal Y Hamd 2024<br>Moritz Blumenthal 2024<br>Cristiana Indolfi 2024<br>Cameron Strong 2024<br>Ioannis Sgouralis 2024<br>Tara N Havens 2024<br>Leon Ericsson 2024<br>Lingjie Xie 2024<br>Dalia Martinez 2024<br>Fengxiang Guo 2024<br><br>Charles H Tator 2024<br>Abdoul Aziz Amadou 2024<br>Hyun-Hae Cho 2024<br>Hamid Mokhtari Torshizi 2024<br>Sarah C Creel 2024<br>Corbin Maciel 2024<br>Shehanaz Shaik 2024<br>Katsunori Oyama 2024<br>Qiangwei Peng 2024<br>Simonas Kecorius 2024<br>Yuan Zhang 2024<br>Natalia Agudelo 2024<br>Saritha Saladi 2024<br>Matthew A Nazari 2024<br>Nikos Perdikopanis 2024<br>Susanna Staccioli 2024<br>Sheng Ye 2024<br>Yuyuan Miao 2024<br>F N van der Weijden 2024<br>Miao Yu 2023<br>Junqi Wang 2024<br>Seongil Han 2024<br>Natalia Arias-Trejo 2024<br>Anjun Yu 2024<br>Yi Luo 2024<br>Joud Mar'i 2024<br>Wei Liu 2024<br>Chan Kulatunga Moruzi 2024<br>Tianjiao Zhang 2024<br>Daniela Galatro 2024<br>Mohd Rashid 2024<br>Baneswar Sarker 2024<br>Chirasak Khamfongkhruea 2024<br>Karen Blackmon 2024<br>Jeremy Purcell 2024<br>Benjamin M Rosenberg 2024<br>Deborah Johnson-Shelton 2024<br>Dong Ouyang 2024<br>Xi Wu 2024<br>Jinbo Qu 2024<br>Chen Chen 2024<br>Yinka Oyelese 2024<br>Taeho Kang 2024<br>Yuan Gao 2024<br>Misty C Richards 2024<br>Hiu Mei Chow 2024<br>Heewon Park 2024<br>Gohar Soufi 2024<br>Qiang Lin 2024							
