# Supplementary material for: The Influence of Noise Exposure on Cognitive Function in Children and Adolescents: A Meta-Analysis
Source: NeuroSci. 2025 Mar 4;6(1):22. doi: 10.3390/neurosci6010022 (PMC11944768; doi:10.3390/neurosci6010022)
Supplement: Supplementary file 1 [file neurosci-06-00022-s001.zip › R code S3.pdf]

Supplementary Material S1

R Code

```
#SMD

cont <- metacont (n1, m1, s1, n2, m2, s2, sm="SMD", method.smd="Hedges", studlab = study, data = cont); print(cont, digits =3);

#forest plot

forest(cont, comb.fixed=TRUE, comb.random=TRUE,digits=3,rightcols=c("effect", "ci"));

#metadata

metainf(cont);

#funnel plot

funnel(cont, comb.fixed=TRUE, comb.random=FALSE); text(x = cont$TE, y = cont$seTE, labels = cont$studlab, pos = 4, cex = 1.0)
```
