# Supplementary material for: The Influence of Noise Exposure on Cognitive Function in Children and Adolescents: A Meta-Analysis
Source: NeuroSci. 2025 Mar 4;6(1):22. doi: 10.3390/neurosci6010022 (PMC11944768; doi:10.3390/neurosci6010022)
Supplement: Supplementary file 1 [file neurosci-06-00022-s001.zip › Search S1.pdf]

S1. Comprehensive search strategy for articles to examine 29the effect of noise exposure in the cognition abilities in children.

PubMed Detailed: 2,968
(("noise"[MeSH Terms] OR "noise"[All Fields] OR "noises"[All Fields] OR "noise s"[All Fields] OR "noised"[All Fields] OR "noising"[All Fields] OR ("acoust"[All Fields] OR "acoustical"[All Fields] OR "acoustically"[All Fields] OR "acoustics"[MeSH Terms] OR "acoustics"[All Fields] OR "acoustic"[All Fields]) OR ("sound"[MeSH Terms] OR "sound"[All Fields] OR "sounded"[All Fields] OR "soundings"[All Fields] OR "sounds"[All Fields] OR "sound s"[All Fields] OR "sounding"[All Fields]) OR (("white"[MeSH Terms] OR "white"[All Fields] OR "white people"[MeSH Terms] OR ("white"[All Fields

] AND "people"[All Fields]) OR "white people"[All Fields] OR "whites"[All Fields]) AND ("noise"[MeSH Terms] OR "noise"[All Fields] OR "noises"[All Fields] OR "noise s"[All Fields] OR "noised"[All Fields] OR "noising"[All Fields])) OR ("acoustic stimulation"[MeSH Terms] OR ("acoustic"[All Fields] AND "stimulation"[All Fields]) OR "acoustic stimulation"[All Fields]) OR (("environment"[MeSH Terms] OR "environment"[All Fields] OR "environmental"[All Fields] OR "environmentally"[All Fields] OR "environmentals"[All Fields]) AND ("noise"[MeSH Terms] OR "noise"[All Fields] OR "noises"[All Fields] OR "noise s"[All Fields] OR "noised"[All Fields] OR "noising"[All Fields])) OR (("noise"[MeSH Terms] OR "noise"[All Fields] OR "noises"[All Fields] OR "noise s"[All Fields] OR "noised"[All Fields] OR "noising"[All Fields]) AND ("stress"[All Fields] OR "stressed"[All Fields] OR "stresses"[All Fields] OR "stressful"[All Fields] OR "stressfulness"[All Fields] OR "stressing"[All Fields])) OR (("chronic"[All Fields] OR "chronical"[All Fields] OR "chronically"[All Fields] OR "chronicities"[All Fields] OR "chronicity"[All Fields] OR "chronicization"[All Fields] OR "chronics"[All Fields]) AND ("noise"[MeSH Terms] OR "noise"[All Fields] OR "noises"[All Fields] OR "noise s"[All Fields] OR "noised"[All Fields] OR "noising"[All Fields])) OR (("acute"[All Fields] OR "acutely"[All Fields] OR "acutes"[All Fields]) AND ("noise"[MeSH Terms] OR "noise"[All Fields] OR "noises"[All Fields] OR "noise s"[All Fields] OR "noised"[All Fields] OR "noising"[All Fields])) OR (("broadband"[All Fields] OR "broadbands"[All Fields]) AND ("noise"[MeSH Terms] OR "noise"[All Fields] OR "noises"[All Fields] OR "noise s"[All Fields] OR "noised"[All Fields] OR "noising"[All Fields]))) AND ("memories"[All Fields] OR "memory"[MeSH Terms] OR "memory"[All Fields] OR "memory s"[All Fields] OR ("spatial memory"[MeSH Terms] OR ("spatial"[All Fields] AND "memory"[All Fields]) OR "spatial memory"[All Fields]) OR ("learning"[MeSH Terms] OR "learning"[All Fields] OR "learn"[All Fields] OR "learned"[All Fields] OR "learning s"[All Fields] OR "learnings"[All Fields] OR "learns"[All Fields]) OR ("memory, short term"[MeSH Terms] OR ("memory"[All Fields] AND "short term"[All Fields]) OR "short-term memory"[All Fields] OR ("memory"[All Fields] AND "short"[All Fields] AND "term"[All Fields]) OR "memory short term"[All Fields]) OR ("episode"[All Fields] OR "episodes"[All Fields] OR "episodic"[All Fields] OR "episodically"[All Fields])) AND ("child"[MeSH Terms] OR "child"[All Fields] OR "children"[All Fields] OR "child s"[All Fields] OR "children s"[All Fields] OR "childrens"[All Fields] OR "childs"[All Fields] OR ("minority groups"[MeSH Terms] OR ("minority"[All Fields] AND "groups"[All Fields]) OR "minority groups"[All Fields] OR "minorities"[All Fields] OR "minority"[All Fields] OR "minority s"[All Fields] OR "minors"[MeSH Terms] OR "minors"[All Fields] OR "minor"[All Fields]) OR

("child"[MeSH Terms] OR "child"[All Fields] OR "children"[All Fields] OR "child s"[All Fields] OR "children s"[All Fields] OR "childrens"[All Fields] OR "childs"[All Fields]) OR ("adolescent"[MeSH Terms] OR "adolescent"[All Fields] OR "youth"[All Fields] OR "youths"[All Fields] OR "youth s"[All Fields]) OR "kids"[All Fields] OR ("young"[All Fields] OR "youngs"[All Fields]))) NOT ("review"[Publication Type] OR "review literature as topic"[MeSH Terms] OR "review"[All Fields] OR ("review"[Publication Type] OR "review literature as topic"[MeSH Terms] OR "review literature"[All Fields]) OR ("meta analysis"[Publication Type] OR "meta analysis as topic"[MeSH Terms] OR "meta analysis"[All Fields]) OR ("systematic review"[Publication Type] OR "systematic reviews as topic"[MeSH Terms] OR "systematic review"[All Fields]) OR ("adult"[MeSH Terms] OR "adult"[All Fields] OR "adults"[All Fields] OR "adult s"[All Fields]) OR ("speech"[MeSH Terms] OR "speech"[All Fields] OR "speeches"[All Fields]) OR ("vowel"[All Fields] OR "vowel s"[All Fields] OR "voweled"[All Fields] OR "vowels"[All Fields]) OR ("rats"[MeSH Terms] OR "rats"[All Fields] OR "rat"[All Fields]) OR ("rats"[MeSH Terms] OR "rats"[All Fields]) OR ("mice"[MeSH Terms] OR "mice"[All Fields] OR "murine"[All Fields] OR "muridae"[MeSH Terms] OR "muridae"[All Fields] OR "murines"[All Fields] OR "murin"[All Fields]) OR ("mice"[MeSH Terms] OR "mice"[All Fields]) OR ("mice"[MeSH Terms] OR "mice"[All Fields] OR "mouse"[All Fields] OR "mouse s"[All Fields] OR "mouses"[All Fields]) OR ("animals"[MeSH Terms:noexp] OR "animal"[All Fields]))

Translations

noise: "noise"[MeSH Terms] OR "noise"[All Fields] OR "noises"[All Fields] OR "noise's"[All Fields] OR "noised"[All Fields] OR "noising"[All Fields]

acoustic: "acoust"[All Fields] OR "acoustical"[All Fields] OR "acoustically"[All Fields] OR "acoustics"[MeSH Terms] OR "acoustics"[All Fields] OR "acoustic"[All Fields]

sound: "sound"[MeSH Terms] OR "sound"[All Fields] OR "sounded"[All Fields] OR "soundings"[All Fields] OR "sounds"[All Fields] OR "sound's"[All Fields] OR "sounding"[All Fields]

white: "white"[MeSH Terms] OR "white"[All Fields] OR "white people"[MeSH Terms] OR ("white"[All Fields] AND "people"[All Fields]) OR "white people"[All Fields] OR "whites"[All Fields]

noise: "noise"[MeSH Terms] OR "noise"[All Fields] OR "noises"[All Fields] OR "noise's"[All Fields] OR "noised"[All Fields] OR "noising"[All Fields]

Acoustic Stimulation: "acoustic stimulation"[MeSH Terms] OR ("acoustic"[All Fields] AND "stimulation"[All Fields]) OR "acoustic stimulation"[All Fields]

environmental: "environment"[MeSH Terms] OR "environment"[All Fields] OR "environmental"[All Fields] OR "environmentally"[All Fields] OR "environmentals"[All Fields]

noise: "noise"[MeSH Terms] OR "noise"[All Fields] OR "noises"[All Fields] OR "noise's"[All Fields] OR "noised"[All Fields] OR "noising"[All Fields]

noise: "noise"[MeSH Terms] OR "noise"[All Fields] OR "noises"[All Fields] OR "noise's"[All Fields] OR "noised"[All Fields] OR "noising"[All Fields]

stress: "stress"[All Fields] OR "stressed"[All Fields] OR "stresses"[All Fields] OR "stressful"[All Fields] OR "stressfulness"[All Fields] OR "stressing"[All Fields]

chronic: "chronic"[All Fields] OR "chronical"[All Fields] OR "chronically"[All Fields] OR "chronicities"[All Fields] OR "chronicity"[All Fields] OR "chronicization"[All Fields] OR "chronics"[All Fields]

noise: "noise"[MeSH Terms] OR "noise"[All Fields] OR "noises"[All Fields] OR "noise's"[All Fields] OR "noised"[All Fields] OR "noising"[All Fields]

acute: "acute"[All Fields] OR "acutely"[All Fields] OR "acutes"[All Fields]

noise: "noise"[MeSH Terms] OR "noise"[All Fields] OR "noises"[All Fields] OR "noise's"[All Fields] OR "noised"[All Fields] OR "noising"[All Fields]

broadband: "broadband"[All Fields] OR "broadbands"[All Fields]

noise: "noise"[MeSH Terms] OR "noise"[All Fields] OR "noises"[All Fields] OR "noise's"[All Fields] OR "noised"[All Fields] OR "noising"[All Fields]

memory: "memories"[All Fields] OR "memory"[MeSH Terms] OR "memory"[All Fields] OR "memory's"[All Fields]

spatial memory: "spatial memory"[MeSH Terms] OR ("spatial"[All Fields] AND "memory"[All Fields]) OR "spatial memory"[All Fields]

learning: "learning"[MeSH Terms] OR "learning"[All Fields] OR "learn"[All Fields] OR "learned"[All Fields] OR "learning's"[All Fields] OR "learnings"[All Fields] OR "learns"[All Fields]

memory short-term: "memory, short-term"[MeSH Terms] OR ("memory"[All Fields] AND "short-term"[All Fields]) OR "short-term memory"[All Fields] OR ("memory"[All Fields] AND "short"[All Fields] AND "term"[All Fields]) OR "memory short term"[All Fields]

Episodic: "episode"[All Fields] OR "episodes"[All Fields] OR "episodic"[All Fields] OR "episodically"[All Fields]

Child: "child"[MeSH Terms] OR "child"[All Fields] OR "children"[All Fields] OR "child's"[All Fields] OR "children's"[All Fields] OR "childrens"[All Fields] OR "childs"[All Fields]

Minors: "minority groups"[MeSH Terms] OR ("minority"[All Fields] AND "groups"[All Fields]) OR "minority groups"[All Fields] OR "minorities"[All Fields] OR "minority"[All Fields] OR "minority's"[All Fields] OR "minors"[MeSH Terms] OR "minors"[All Fields] OR "minor"[All Fields]

children: "child"[MeSH Terms] OR "child"[All Fields] OR "children"[All Fields] OR "child's"[All Fields] OR "children's"[All Fields] OR "childrens"[All Fields] OR "childs"[All Fields]

Youths: "adolescent"[MeSH Terms] OR "adolescent"[All Fields] OR "youth"[All Fields] OR "youths"[All Fields] OR "youth's"[All Fields]

young: "young"[All Fields] OR "youngs"[All Fields]

review: "review"[Publication Type] OR "review literature as topic"[MeSH Terms] OR "review"[All Fields]

Review Literature: "review"[Publication Type] OR "review literature as topic"[MeSH Terms] OR "review literature"[All Fields]

Meta-Analysis: "meta-analysis"[Publication Type] OR "meta-analysis as topic"[MeSH Terms] OR "meta-analysis"[All Fields]

systematic review: "systematic review"[Publication Type] OR "systematic reviews as topic"[MeSH Terms] OR "systematic review"[All Fields]

adults: "adult"[MeSH Terms] OR "adult"[All Fields] OR "adults"[All Fields] OR "adult's"[All Fields]

Speech: "speech"[MeSH Terms] OR "speech"[All Fields] OR "speech's"[All Fields] OR "speeches"[All Fields]

vowel: "vowel"[All Fields] OR "vowel's"[All Fields] OR "voweled"[All Fields] OR "vowels"[All Fields]

rat: "rats"[MeSH Terms] OR "rats"[All Fields] OR "rat"[All Fields]

rats: "rats"[MeSH Terms] OR "rats"[All Fields]

murine: "mice"[MeSH Terms] OR "mice"[All Fields] OR "murine"[All Fields] OR "muridae"[MeSH Terms] OR "muridae"[All Fields] OR "murines"[All Fields] OR "murin"[All Fields]

mice: "mice"[MeSH Terms] OR "mice"[All Fields]

mouse: "mice"[MeSH Terms] OR "mice"[All Fields] OR "mouse"[All Fields] OR "mouse's"[All Fields] OR "mouses"[All Fields]

animal: "animals"[MeSH Terms:noexp] OR animal[All Fields]

Web of Science: 2,158

TS=((noise OR noises OR acoustic OR acoustics OR sound OR "white noise" OR "acoustic stimulation" OR "environmental noise" OR "noise stress" OR "chronic noise" OR "acute noise" OR "broadband noise") AND (memory OR "spatial memory" OR learning OR "short-term memory" OR "episodic memory") AND (child OR children OR adolescents OR youth OR kids OR minors))

NOT TS= (review OR "meta-analysis" OR "systematic review" OR adult OR speech OR vowel OR rats OR mice OR animals)
